# FaMYB44.2, a transcriptional repressor, negatively regulates sucrose accumulation in strawberry receptacles through interplay with FaMYB10

**DOI:** 10.1093/jxb/ery249

**Published:** 2018-08-02

**Authors:** Lingzhi Wei, Wenwen Mao, Meiru Jia, Sinian Xing, Usman Ali, Yaoyao Zhao, Yating Chen, Minglin Cao, Zhengrong Dai, Kai Zhang, Zhechao Dou, Wensuo Jia, Bingbing Li

**Affiliations:** College of Horticulture, China Agricultural University, Beijing, China

**Keywords:** FaMYB44.2, FaMYB10, FaSPS3, jasmonic acid, strawberry receptacles, sucrose accumulation

## Abstract

Sugar and acid metabolism are critical for fruit ripening and quality formation, but the underlying regulatory mechanisms are largely unknown. Here, we identified a transcriptional repressor, FaMYB44.2, that regulates sugar and acid accumulation in strawberry (*Fragaria × ananassa* ‘Benihoppe’) receptacles. We transiently expressed *FaMYB44.2* in strawberry fruit and conducted metabolic and molecular analyses to explore the role of FaMYB44.2 in sugar and acid accumulation in strawberry. We found that FaMYB44.2 negatively regulates soluble sugar accumulation and malic acid content and represses the expression of numerous structural genes, including *FaSPS3*, a key gene in sucrose accumulation. From the white fruit stage onwards, the repressive effect of FaMYB44.2 on *FaSPS3* is reversed by FaMYB10, which positively regulates anthocyanin accumulation. Our results indicate that FaMYB10 suppresses *FaMYB44.2* expression; weakens the interaction between FaMYB44.2 and its co-repressor, FabHLH3; and cooperates with FabHLH3 to activate the expression of *FaSPS3*. The interplay between FaMYB10 and FaMYB44.2 results in sucrose accumulation in ripe strawberry fruits. In addition, the repressive effect of FaMYB44.2 on sucrose accumulation is enhanced by jasmonic acid. This study provides new insights into the regulatory mechanisms of sucrose accumulation and sheds light on the interplay between regulatory proteins during strawberry fruit ripening and quality formation.

## Teaser

FaMYB44.2 is a novel transcriptional repressor that modulates both ripening-related and jasmonic acid-related sucrose accumulation in strawberry receptacles.

## Introduction

Strawberry (*Fragaria × ananassa* ‘Benihoppe’), an important horticultural crop worldwide, has excellent commercial value and health benefits (Sturzeanu *et al.*, 2013). Sugar content in strawberry fruit is mainly attributed to the accumulation of glucose, fructose, and sucrose (Fait *et al.*, 2008; Ogiwara *et al.*, 2008). During fruit development and ripening, glucose and fructose content in strawberry receptacles increase steadily, but the sucrose content remains low during early fruit development and rises sharply from the white fruit stage onwards (Ogiwara *et al.*, 2008; Jia *et al.*, 2013). During fruit ripening, sucrose levels can increase up to 25-fold, whereas glucose and fructose levels increase only ~2-fold (Fait *et al.*, 2008; Merchante *et al.*, 2013; Vallarino *et al.*, 2015); thus, sucrose accumulation during fruit ripening is a key determinant of quality in strawberry fruit. Sucrose also functions as an important signaling molecule that regulates anthocyanin accumulation in strawberry fruit through *FaSUT1*-mediated abscisic acid (ABA) signaling; *FaSUT1* is a sucrose transporter that regulates sucrose transport from leaves to fruit via long-distance transport in the phloem (Jia *et al.*, 2013). However, the mechanisms regulating sucrose accumulation and sucrose signal transduction in strawberry fruit are unclear.

Studies investigating fruit ripening in strawberry have mainly focused on the mechanisms regulating anthocyanin accumulation (Jiang and Joyce, 2003; Medina-Puche *et al.*, 2014; Lin-Wang *et al.*, 2014; Kadomura-Ishikawa *et al.*, 2015). Several important regulatory proteins involved in anthocyanin accumulation have been identified, such as the protein kinases FaSnRK2.6 and FaMRLK47 and the transcription factors FaMYB1 and FaMYB10 (Aharoni *et al.*, 2001; Medina-Puche *et al.*, 2014; Lin-Wang *et al.*, 2014; Han *et al.*, 2015; Kadomura-Ishikawa *et al.*, 2015; Jia *et al.*, 2017). We recently found that FaMRLK47, a negative regulator of anthocyanin accumulation, also functions as a positive regulator of sugar accumulation and that two important structural genes involved in sucrose accumulation, *FaSPS3* (sucrose-6-phosphate synthase 3, *gene31122*) and *FaSUS1* (sucrose synthase 1, *gene12940*), are key structural genes downstream of FaMRLK47 (Jia *et al.*, 2017).

Vallarino *et al.* (2015) also found that *FaSPS3* (*gene31122*) and *FaSUS1* (*gene12940*) contribute to ripening-related sucrose accumulation and are down-regulated by transient silencing of *FaGAMYB*, a gibberellin (GA)-related MYB transcription factor that plays a central role in the transition of strawberry receptacles from developing to ripening. Furthermore, silencing of *FaGAMYB* decreased both sucrose content and the expression level of the positive anthocyanin biosynthesis regulatory gene *FaMYB10* (Vallarino *et al.*, 2015). However, unexpectedly, in *FaMRLK47*-overexpression (OE) and *FaMRLK47*-RNAi fruits, *FaMYB10* was up-regulated and down-regulated, respectively (Jia *et al.*, 2017). Additionally, RNA-sequence analysis of *FvMYB10*-RNAi and *FvMYB10*-OE transgenic fruits showed that various transcription factor genes not associated with anthocyanin accumulation, such as *FvMYB89* (named *FaEBOII* in octoploid strawberry), are also regulated by FvMYB10 (Lin-Wang *et al.*, 2014; Medina-Puche *et al.*, 2015). Together, these findings suggest that in addition to regulating anthocyanin biosynthesis, MYB10 plays diverse roles in regulating strawberry fruit ripening. Transient silencing of *FaMYB10* in strawberry fruits also reduced the expression of *FaSPS1* (*gene11606*) and *FaHXK* (*gene25718*) (Medina-Puche *et al.*, 2015); *FaSPS1* is involved in sucrose accumulation and *HXK* may be related to both sugar signaling transduction and sugar metabolism regulation (Rolland *et al.*, 2006; Chai *et al.*, 2011). We hypothesized that FaMYB10 functions in the regulation of sucrose content. However, this hypothesis has yet to be tested.

In plants, MYB transcription factors are classified into the R1-MYB, R2R3-MYB, and R1R2R3-MYB subfamilies according to the number of conserved MYB structural motifs at their N-termini (Du *et al.*, 2009; Dubos *et al.*, 2010). Most MYB transcription factors in plants are R2R3-MYB transcription factors, which are widely involved in the regulation of plant growth, hormone signal transduction, stress and disease resistance, and secondary metabolism (Kranz *et al.*, 1998; Stracke *et al.*, 2001; Jiang *et al.*, 2004). To perform their biological functions, MYB transcription factors frequently couple with basic helix–loop–helix proteins (bHLHs) and TTG1 (WD40 protein) to form a ternary MYB–bHLH–WD40 (MBW) complex (Schaart *et al.*, 2013). The functions of MYB can be affected by their MBW partners at various levels (Gonzalez *et al.*, 2008; Hichri *et al.*, 2011; Petroni and Tonelli, 2011; Schaart *et al.*, 2013; Li, 2014). The transcriptional activities of MBW complexes can also be regulated by other transcription factors (Dubos *et al.*, 2008; Matsui *et al.*, 2008; Qi *et al.*, 2011; Xie *et al.*, 2016).

In strawberry, MYB transcription factors comprise a large family, including 111 MYB proteins and 61 MYB-related proteins (Zheng *et al.*, 2016). The functions of most MYBs in strawberry are unclear, but several are involved in regulating proanthocyanin, flavonoid, and anthocyanin accumulation in fruits (Aharoni *et al.*, 2001; Medina-Puche *et al.*, 2014; Lin-Wang *et al.*, 2014; Kadomura-Ishikawa *et al.*, 2015). FaMYB10 and FaMYB1 function as positive and negative regulators of anthocyanin and flavonoid accumulation, respectively, and FaMYB10 and FaMYB1 might couple with FabHLH3/33 (*gene08231*/*gene19321*) and/or FaTTG1 (WD40 protein, *gene12450*) to form a ternary MBW complex that regulates anthocyanin and flavonoid accumulation (Schaart *et al.*, 2013). The bHLH subunits might play redundant roles in FaMYB10-modulated MBW complex formation (Schaart *et al.*, 2013; Lin-Wang *et al.*, 2014), but only one gene (*FaTTG1*, *gene12450*) is present in the strawberry genome for the WD40 subunit of this complex (Schaart *et al.*, 2013). However, the regulatory roles of the different subunits in these complexes and the specific members involved require further exploration.

Sucrose accumulation is affected by multiple signals, including light, hormones, and stresses (Vassey, 1989; Guy *et al.*, 1992; Huber *et al.*, 1992; Pérez *et al.*, 1997; Jia *et al.*, 2011). ABA and jasmonic acid (JA) might be involved in regulating sucrose accumulation in strawberry fruits (Pérez *et al.*, 1997; Jia *et al.*, 2011); however, little is known about the hormone-mediated changes in sugar content in strawberry fruits. The identification of regulatory proteins, such as transcription factors related to fruit ripening or hormone signal transduction, will provide important clues about the regulatory mechanisms of strawberry fruit ripening and quality formation. MYB44 is an important transcription factor in various species, including Arabidopsis, apple (*Malus domestica*), and potato (*Solanum tuberosum* L.) (Jung *et al.*, 2008; Persak and Pitzschke, 2013; Jaradat *et al.*, 2013; Zhao *et al*., 2016*b*; Wu *et al.*, 2017; Zhou *et al.*, 2017). Transgenic analysis showed that MYB44 regulates plant growth, stress resistance, and hormone signal transduction (Jung *et al.*, 2008; Jaradat *et al.*, 2013; Shim *et al.*, 2013; Persak and Pitzschke, 2014); however, the roles of MYB44 in fruit ripening have not been reported.

In this study, we identified FaMYB44.2, an R2R3 MYB transcriptional repressor in strawberry fruit. Transient transgenic analysis showed that FaMYB44.2 regulates soluble sugar and organic acid content in strawberry fruits. Combining the results of metabolic and molecular experiments, we further revealed that FaMYB44.2 regulates sucrose accumulation through repressing *FaSPS3* expression. As fruit ripening progressed, the highly expressed FaMYB10 reversed its repressive effect on *FaSPS3*, ultimately resulting in sucrose accumulation during the late ripening stages. Additionally, we demonstrated that FaMYB44.2 is involved in JA-repressed sucrose accumulation in strawberry fruits. The results of this study shed light on the transcriptional regulatory mechanisms underlying ripening-related and hormone-mediated sucrose accumulation in strawberry fruits.

## Materials and methods

### Plant materials and growth conditions

Strawberry plants (*Fragaria × ananassa* ‘Benihoppe’) were grown in a greenhouse under controlled conditions under conditions of 16 h/8 h (day/night) photoperiod at 450 µmol m^−2^ s^−1^ and 65% humidity, with a day/night temperature of 25 °C/15 °C. Fruit ripening stages were classified based on days post-anthesis (DPA) (Jia *et al.*, 2013).

### Gene isolation and sequence alignment

To identify *MYB44s* in the strawberry genome, the protein sequence of AtMYB44 (AT5G67300) was used as a query for BLAST analysis against the NCBI database (http://www.ncbi.nlm.nih.gov/) and the strawberry genome database (http://strawberry-garden.kazusa.or.jp/index.html). Three *MYB44* genes were isolated from strawberry receptacles. Multiple alignments of FaMYB44 and FaMYB1 were carried out using Clustal X 2.0.12 (Thompson *et al.*, 1997). The alignment results were edited and marked using GeneDoc. A phylogenetic tree of FaMYB1 and MYB44s in different species was constructed via the neighbor-joining method using MEGA7 software with 1000 bootstrap replicates to evaluate the reliability of different phylogenetic groups. The full-length protein sequences of MYB44s and FaMYB1 are provided in Supplementary Table S1 at *JXB* online.

### Transient transformation of strawberry fruits by agroinfiltration

Strawberry fruits were transformed via agroinfiltration as previously described (Hoffmann *et al.*, 2006). The Gateway vector pH7W2GD and the RNAi vector pFGC5941 were used to overexpress and silence *FaMYB44.2*, respectively, while pBI121 was used to overexpress *FaMYB10*. *FaMYB10*-OE, *FaMYB44.2*-OE, *FaMYB44.2*-RNAi, and empty vectors (EV-OE and EV-RNAi) were transformed individually into *Agrobacterium tumefaciens* strain GV3101 and grown at 28 °C in LB liquid medium containing the appropriate antibiotics. When the OD_600_ of the culture reached approximately 0.8, the agrobacterium cells were centrifuged and resuspended in infiltration buffer [10 mM MgCl_2_, 10 mM MES (pH 5.6), and 100 µM acetosyringone] and shaken for 2 h at room temperature before being used for infiltration. To identify the function of FaMYB44.2 during fruit ripening, 30 white fruits were infiltrated per construct, and 120 fruits were used per experiment. After infiltration, controls, *FaMYB44.2*-OE, and *FaMYB44.2*-RNAi infiltrated fruits were photographed every 3 d to record their phenotypes, and EV-OE, *FaMYB44.2*-OE, EV-RNAi and *FaMYB44.2*-RNAi fruits were collected at 9 d after infiltration to analyse ripening-related parameters and the expression of ripening-related genes. For *FaMYB10*-OE, 30 detached fruits were infiltrated with *FaMYB10*-OE or control vector in a single experiment, and the samples were collected for ripening-related gene expression analysis at 5 d after infiltration. All fruits were frozen in liquid nitrogen and, after removing the achenes, the receptacles were stored at −80 °C until further analysis. For each individual experiment, three biological repeats were performed. The primers are provided in Supplementary Tables S2, S3.

### Measurement of fruit ripening-associated physiological parameters

Fruit firmness was measured using a GY-4 fruit hardness tester (Zhejiang TOP Instrument) with a 3 mm probe. The contents of specific sugar and acid components, as well as other fruit ripening-related parameters listed in Table 1, were measured by HPLC as described by Han *et al.* (2015) and Kafkas *et al.* (2007). For each parameter, 30 fruits were used per sample and three individual biological replicates were performed.

**Table 1.** Effects of *FaMYB44.2*-OE and *FaMYB44.2*-RNAi on the major fruit ripening-related parameters

Parameter	EV-OE	OE	EV-RNAi	RNAi
Firmness^*a*^ (kg cm^−2^)	4.76 ± 0.86	5.20 ± 1.67	5.09 ± 0.77	5.18 ± 0.55
Flavonoid content^*b*^ (μg g^−1^ fresh wt)	3.11 ± 0.62	2.82 ± 0.66	2.95 ± 0.81	3.27 ± 0.93
Anthocyanin content^*b*^ (mg g^−1^ fresh wt)	0.54 ± 0.23	0.33 ± 0.10	0.56 ± 0.35	0.61 ± 0.07
Total phenol content^*b*^ (mg g^−1^ fresh wt)	3.97 ± 0.09	4.25 ± 0.33	3.78 ± 0.04	3.51 ± 0.11
Aroma metabolism-related compounds^*c*^				
Acetic acid, hexyl ester	2.13 ± 0.52	1.54 ± 0.28	2.38 ± 0.29	2.07 ± 0.02
Hexanal	22.6 ± 2.29	22.2 ± 4.6	22.1 ± 2.62	23.9 ± 1.36
2-Hexenal (E)	45.5 ± 1.2	52.5 ± 1.1^∗^	43.1 ± 1.1	54.7 ± 2.4^∗^
2-Hexen-1-ol (E)	1.59 ± 0.31	1.17 ± 0.38	1.42 ± 0.35	1.27 ± 0.22
1-Hexanol	1.17 ± 0.18	1.08 ± 0.36	1.07 ± 0.17	0.61 ± 0.20^∗^
Butanoic acid, methyl ester	1.65 ± 0.04	1.28 ± 0.28	1.55 ± 0.22	0.40 ± 0.06^∗∗^
Furan, 2-pentyl	0.11 ± 0.03	0.24 ± 0.01	0.14 ± 0.02	0.15 ± 0.09
2,3-Octanedione	0.16 ± 0.03	0.08 ± 0.01	0.21 ± 0.05	0.22 ± 0.04
Hexanoic acid, methyl ester	0.53 ± 0.32	1.88 ± 0.74^∗∗^	0.59 ± 0.62	0.22 ± 0.03^∗^
2-Hexen-1-ol, acetate (E)	3.26 ± 0.86	1.32 ± 0.33^∗^	3.55 ± 0.64	6.34 ± 0.09^∗^
Pentanal	0.51 ± 0.22	0.84 ± 0.23	0.39 ± 0.17	1.29 ± 0.45^∗^
2-Pentenal (E)	0.18 ± 0.07	0.28 ± 0.09	0.16 ± 0.16	0.71 ± 0.02^∗∗^
1-Pentanol	0.21 ± 0.03	0.37 ± 0.16	0.23 ± 0.26	0.76 ± 0.05^∗^
2-Heptenal (Z)	0.31 ± 0.02	0.34 ± 0.11	0.35 ± 0.07	0.68 ± 0.03^∗^
Benzaldehyde	0.23 ± 0.10	0.16 ± 0.07	0.24 ± 0.03	0.17 ± 0.02
1-Penten-3-one	0.33 ± 0.07	0.77 ± 0.03^∗^	0.37 ± 0.01	0.09 ± 0.01^∗^
Nonanal	0.26 ± 0.11	0.19 ± 0.03	0.24 ± 0.08	0.21 ± 0.06
Octanal	0.23 ± 0.03	0.11 ± 0.04	0.25 ± 0.01	0.12 ± 0.02
1,6-Octadien-3-ol, 3,7-dimethyl	0.23 ± 0.01	3.06 ± 1.19^∗∗^	0.29 ± 1.01	0.27 ± 0.05

The *FaMYB44.2*-OE and *FaMYB44.2*-RNAi constructs were transiently expressed in strawberry fruits at white fruit stage, and 9 d after transfection, fruits were detached and analysed. Values are means ±SD of three samples (each sample includes thirty fruits). Student’s *t*-test was used to determine significant differences compared with the control: *P<0.05, **P<0.01. EV-OE, control samples for overexpression; EV-RNAi, control samples for interference; OE, overexpression; RNAi, RNA interference.

^*a*^ Cell wall metabolism-related parameter.

^*b*^ Pigment metabolism-related compounds.

^*c*^ Aroma metabolism-related compounds are expressed as percentage of the total volatiles.

### Detection of protein interactions

#### Yeast two-hybrid assay

A yeast two-hybrid assay (Y2H) was performed using the Matchmaker GAL4-based Two-Hybrid System 3 as described in the *Yeast Protocols Handbook* (Clontech). The full-length cDNA sequences of *FaMYB44.1*, *FaMYB44.2*, *FaMYB44.3*, *FabHLH3*, *FabHLH33*, *FaTTG1*, *FaMYB10*, and FaMYB1 were cloned into the pGADT7 or pGBKT7 vector (see Supplementary Table S2), and the prey and bait constructs were co-transformed into yeast strain AH109 via the lithium acetate method. The interaction of each co-transformation combination was verified by growing co-transformants on minimal −Leu/−Trp/−His (SD-3) medium and −Leu/−Trp/−His/−adenine (SD-4) medium containing 20 mg ml^−1^ X-gal.

#### Bimolecular fluorescence complementation

Constructs expressing the YFP^N^ and YFP^C^ fusions were created using pSPYNE and pSPYCE (see Supplementary Table S2) and co-transformed into tobacco leaves as described previously (Schütze *et al.*, 2009). The full-length coding sequence (CDS) of *FaMYB44.2* was cloned into PMDC83 (Supplementary Table S2) and transformed into tobacco leaves to observe the subcellular localization of the fusion proteins. The infiltrated tobacco leaves were incubated at 23 °C under an 8 h/16 h light/dark cycle for 72 h and subjected to green fluorscent protein (GFP) fluorescence observation under a confocal microscope (Olympus Fluoview FV1000).

#### Firefly luciferase complementation imaging assay

The FaMYB44.2-nLUC, FabHLH3-cLUC, and FaTTG1-cLUC constructs were produced using pCAMBIA1300-cLUC (Chen *et al*., 2008; Supplementary Table S2), and *Agrobacterium* strains harboring the nLUC or cLUC constructs were co-transformed at a 1:1 ratio into strawberry fruits by agroinfiltration as described previously (Ren *et al.*, 2018). Luciferase activity was observed using Lumazone with Winview software.

#### Competitive binding assay

The CDS of FaMYB10 was cloned into pBI121 to generate the *FaMYB10*-OE construct (see Supplementary Table S4), which was co-transformed with FaMYB44.2-nLUC and FabHLH3-cLUC into tobacco leaves and strawberry fruits (Chen *et al*., 2008). Luciferase activity was observed using Lumazone with Winview software.

### Electrophoretic mobility shift assay

The CDS region of *FaMYB44.2* was cloned into the pGEX4T-1 vector to generate a glutathione *S*-transferase (GST) fusion protein (see Supplementary Table S2). The construct was transformed into *Escherichia coli* BL21(DE3) cells and purified using glutathione Sepharose beads (GE Healthcare) as previously described (Xu *et al.*, 2015). Oligonucleotide probes of structural genes were synthesized and labeled with 5′-biotin (Sangon Biotech) by annealing complementary oligonucleotides at 72 °C for 30 min (Supplementary Table S5). The electrophoretic mobility shift assay (EMSA) was performed using a LightShift^®^ Chemiluminescent EMSA Kit (Thermo Fisher Scientific) according to the manufacturer’s instructions.

### Methyl jasmonate treatment

Detached strawberry fruits were transformed with various vectors by agroinfiltration, followed by incubation at 23 °C, 80% humidity under a 12 h/12 h light/dark cycle for 4 d to express the target genes. The transgenic and control fruits were then sprayed with 100 μM methyl jasmonate (MeJA) (Sigma-Aldrich, MKBT7772V) or with an equivalent amount of water and further incubated under the same conditions for 6 h for the gene expression analysis, 6 h for the β-glucuronidase (GUS) activity assay, and 48 h for the sugar content analysis. For gene expression analysis and sugar content analysis, 30 fruits were used per experiment, with three individual experimental repeats; for GUS activity assay, five fruits were used per experiment, and three biological experiments were performed. The primers are listed in Supplementary Table S3.

### GUS activity assay

The promoter sequence of *FaSPS3* (named P_*FaSPS3*_, 1500 bp upstream of the ATG start codon) was cloned into pCAMBIA1304 to generate the reporter construct pCAMBIA1304-P_*FaSPS3*_::GUS. The CDS regions of *FaMYB44.2*, *FabHLH3*, and *FaTTG1* were cloned into pCAMBIA1304-P_*FaSPS3*_::GUS to generate effector constructs and then the constructs were transformed into strawberry fruits by agroinfiltration to evaluate the effects of various effectors on GUS activity. The CDS of *FaMYB10* or *FaMADS9* was cloned into pBI121 or pSPYCE to generate an effector construct and was then co-transformed with different pCAMBIA1304-P_*FaSPS3*_::GUS constructs into strawberry fruits by agroinfiltration to evaluate the effect of FaMYB10 on GUS activity. Agroinfiltration was performed as described above. GUS activity was measured as described by Xie *et al.* (2012). For each experiment, five fruits were infiltrated and combined into one sample, and three experiments were performed. The primers and enzyme sites are listed in Supplementary Table S4.

### Analysis gene expression with semi-quantitive PCR (RT-PCR) and quantitative real time PCR (qRT-PCR)

Total RNA or total micro RNA was extracted from strawberry receptacles using an E.Z.N.A.^®^ Total RNA Kit (Omega) or an EASYspin plant microRNA isolation kit (Aidlab, RN4001) according to the manufacturer’s instructions. The cDNA was synthesized from total RNA using M-MLV reverse transcriptase (Promega). Poly(A) modification and first-strand cDNA synthesis were performed with miRcute miRNA first-strand cDNA synthesis kit (Tiangen, KR201). RT-PCR was performed using PCR-mix (Tuoyingfang) following the manufacturer’s instructions. qRT-PCR was performed using SYBR Premix ExTaq (Takara) (Han *et al.*, 2015; Jia *et al.*, 2017) and miRcute miRNA qPCR Detection Kit (Tiangen, FP401). *FaACTIN* was used as an internal control for gene expression, while U6 RNA was used as an internal control for miRNA expression. The 2^−ΔΔ*C*T^ method was used to calculate transcript levels (Schmittgen and Livak, 2008). Three biological replicates were repeated. Error bars are ±SD values of three replicates. Significance was determined by Student’s *t*-test (*P<0.05 and **P<0.01). The primers are listed in Supplementary Tables S3, S6, S7.

### RNA-Seq analysis

Total RNA was extracted from strawberry receptacles (*Fragaria × ananassa* ‘Benihoppe’) using an E.Z.N.A.® Total RNA Kit (Omega). For each developmental sample, 30 fruits were pooled, and three biological replicates were carried out. RNA-seq analysis was performed by Novogene Co. (Beijing, China) as previously described (Preuß *et al.*, 2014). The reads per kilobase million (RPKM) value was used to present the gene expression level, while the fold change was calculated using the log2 ratio of RPKM value between different samples (see Supplementary Table S8). Illumina reads of all three samples have been submitted to NCBI database (accession number SRP149316; http://www.ncbi.nlm.nih.gov/sra).

### Statistical analysis

All samples were analysed in triplicate, and all data are represented as the mean ±SD. Significance analysis was performed with SPSS software (version 13.0). Statistically significant differences between samples were determined using Student’s *t*-test (*P<0.05, **P<0.01). Multiple comparisons were tested using Turkey’s test and significant differences at the *P*<0.05 level are indicated by different letters.

## Results

### Characterization of FaMYB44 in strawberry

**Fig. 1.**
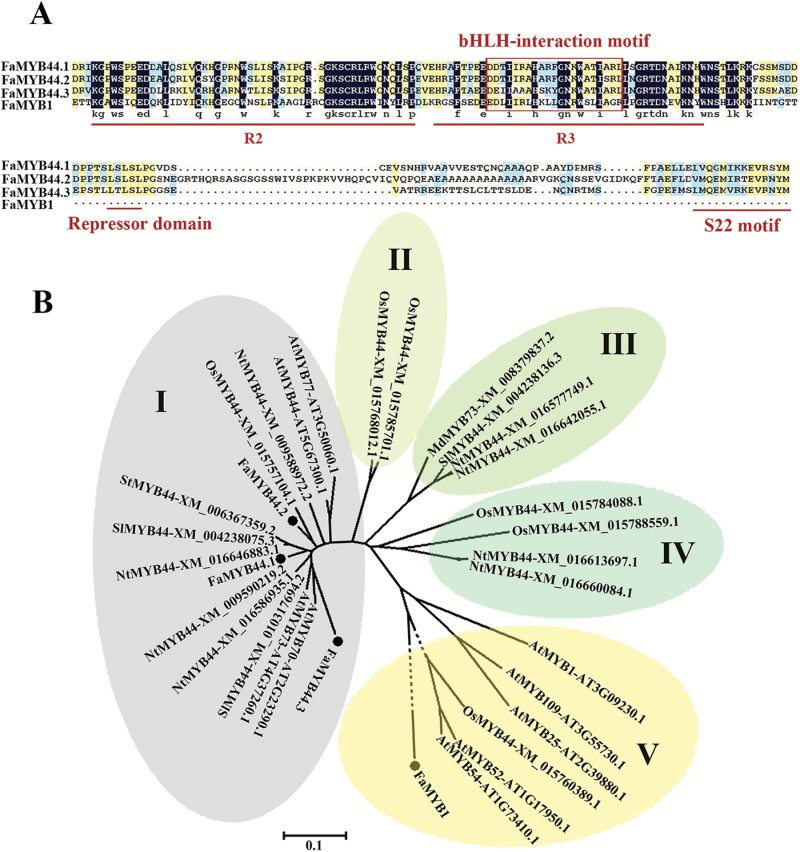
Phylogenetic analysis and multiple sequence alignment of MYB44. (A) Multiple sequence alignment was performed using the protein sequences of three FaMYB44 candidates and FaMYB1; the R2 and R3 domains are labeled and bHLH-interaction motifs are included in the R3 domain. All these three candidates have the conserved domain (GxFMxVV**QEMIxxEVRSYM**) of Arabidopsis MYB subgroup S22 (shown in bold) and the negative repressor motif LxLxL. (B) Phylogenetic analysis of MYB44. At, *Arabidopsis thaliana*; Fa, *Fragaria × ananassa*; Md, *Malus domestica*; Sl, *Solanum lycopersicum*; Nt, *Nicotiana tabacum* L; St, *Solanum tuberosum*; Os, *Oryza sativa*. The scale bars indicate that 10 of every 100 amino acids have differences. The full-length protein sequences are provided in Supplementary Table S1.

AtMYB44 is a typical R2R3 MYB transcription factor, which belongs to MYB subgroup 22 (S22), comprising four members, AtMYB44, AtMYB70, AtMYB73, and AtMYB77 (Stracke *et al.*, 2001; Shin *et al.*, 2007). We used the protein sequence of AtMYB44 as a query to BLAST against the strawberry genome database and performed the multiple sequence alignment to identify FaMYB44, three genes harboring the conserved motif of the S22 subgroup GxFMxVV**QEMIxxEVRSYM** (shown in bold), the transcriptional repressor domain LxLxL, and the bHLH interaction motif (Kranz *et al.*, 1998; Romero *et al.*, 1998; Stracke *et al.*, 2001; Hiratsu *et al.*, 2003; Jia *et al*., 2003; Kagale *et al.*, 2010; Feng *et al.*, 2018; Fig. 1A) were named as FaMYB44.1, FaMYB44.2, and FaMYB44.3. Phylogenetic analysis indicated that FaMYB44.1, FaMYB44.2, and FaMYB44.3 belong in clade I, together with all Arabidopsis S22 subgroup members (Fig. 1B). FaMYB44.1 was previously described as FaMYB76 and FaMYB44.2 as FaMYB40 (Lin-Wang *et al.*, 2014).

**Fig. 2.**
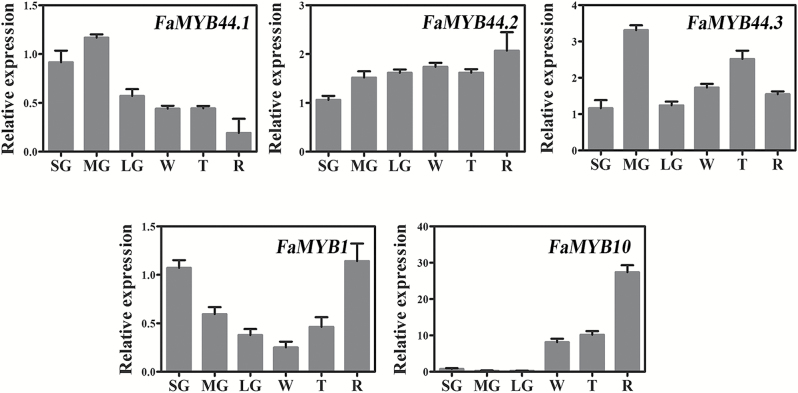
The expression patterns of *FaMYB44*, *FaMYB10*, and *FaMYB1* genes in strawberry fruit at different developmental stages. LG, large green fruit stage; MG, medium green fruit stage; R, fully red stage; SG, small green fruit stage; T, turning stage; W, white fruit stage. Values are means ±SD of three biological replicates normalized using *FaACTIN* as an internal control. The Δ*C*T of small green fruit was chosen as the calibrator (Schmittgen & Livak, 2008).

To investigate the potential roles of FaMYB44s in fruit ripening, we examined the expression levels of *FaMYB44s* in fruits at different developmental stages. As shown in Fig. 2, *FaMYB44.1* expression decreased during fruit development and ripening, whereas *FaMYB44.2* expression slightly increased during fruit ripening. We also quantified the expression of two MYB genes previously shown to regulate strawberry fruit ripening, *FaMYB1* and *FaMYB10*, at different developmental stages, and found their expression patterns to be consistent with those reported previously (Aharoni *et al.*, 2001; Lin-Wang *et al.*, 2014).

### FaMYB44.2 regulates soluble sugar and organic acid accumulation in strawberry fruits

**Fig. 3.**
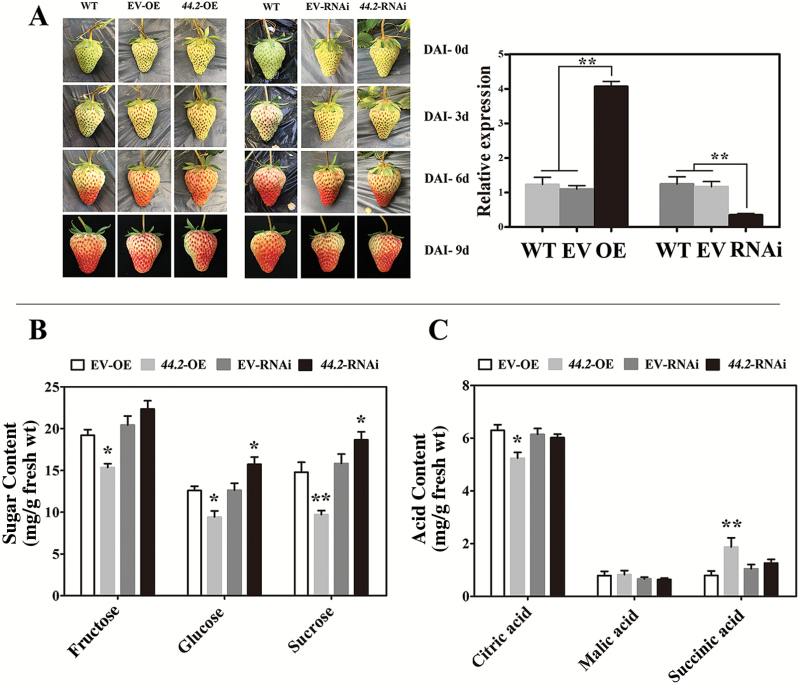
Transient expression of *FaMYB44.2* in strawberry fruits. (A) Phenotypes and expression levels of *FaMYB44.2*-OE and *FaMYB44.2*-RNAi fruits. Expression values are means ±SD of three biological replicates and normalized using *FaACTIN* as an internal control. The Δ*C*T of empty vector (EV) sample was chosen as the calibrator. Statistical significance was determined by Student’s *t*-test: **P<0.01. (B) Sugar content in *FaMYB44.2*-OE and *FaMYB44.2*-RNAi strawberry fruits. (C) Organic acid content in *FaMYB44.2*-OE and *FaMYB44.2*-RNAi strawberry fruits. Statistically significant differences from control were determined by Student’s *t*-test: *P<0.05; **P<0.01. Values are means ±SD of three biological replicates.

To reveal the roles of FaMYB44.1 and FaMYB44.2 in strawberry fruit ripening, we transiently expressed *FaMYB44.1* and *FaMYB44.2* in octoploid strawberry fruits via agroinfiltration. The results suggested that FaMYB44.1 is involved in anthocyanin accumulation (unpublished data), but no obvious differences in anthocyanin accumulation were detected between the control and fruit transiently expressing *FaMYB44.2* (Fig. 3A). Nevertheless, we further analysed changes in the contents of specific sugars and acids in these transgenic fruits, finding that *FaMYB44.2* overexpression significantly reduced the contents of the major sugar components in strawberry fruits, i.e. glucose, sucrose, and fructose (Fig. 3B). However, the changes in organic acid contents differed among components: whereas the citric acid content was lower in *FaMYB44.2*-OE than in the control, the succinic acid content was higher in *FaMYB44.2*-OE, suggesting that the mechanisms regulating *FaMYB44.2*-mediated organic acid accumulation are complex (Fig. 3C). In addition, as shown in Table 1, we also analysed the other ripening-related parameters in *FaMYB44.2* transgenic fruits and found that FaMYB44.2 may be involved in regulating the production of aroma compounds in strawberry fruits.

**Fig. 4.**
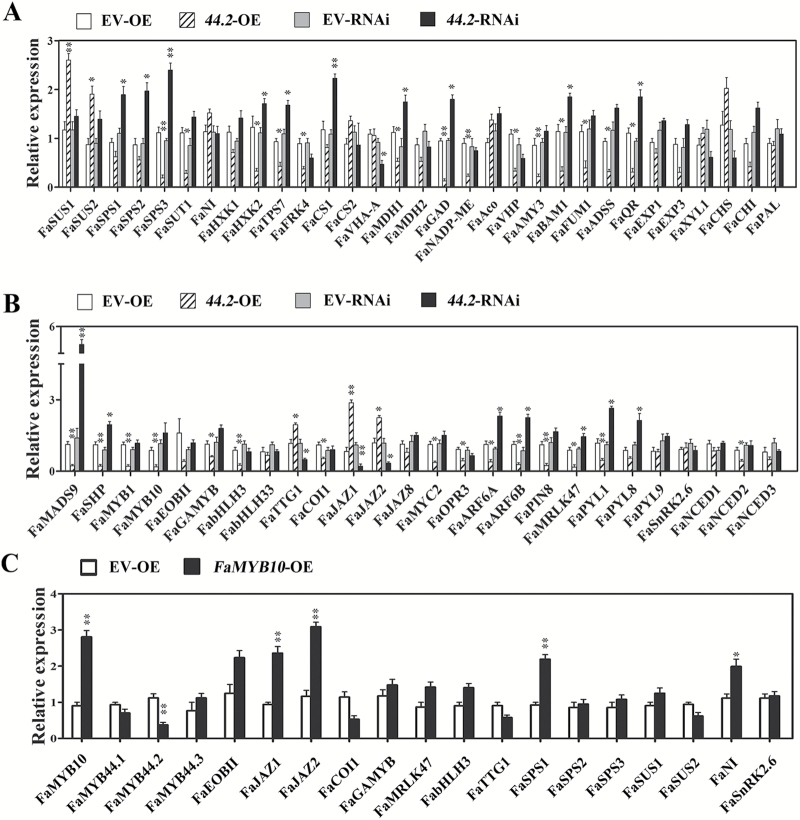
Detecting the expression of ripening-related genes in *FaMYB44.2*-OE and *FaMYB44.2*-RNAi strawberry fruits via qRT-PCR. (A) The expression of ripening-related structural genes in *FaMYB44.2*-OE (*44.2*-OE) and *FaMYB44.2*-RNAi (*44.2*-RNAi) fruits. (B) The expression of regulatory genes in *FaMYB44.2*-OE (*44.2*-OE) and *FaMYB44.2*-RNAi (*44.2*-RNAi) fruits. (C) The expression of fruit ripening-related genes in *FaMYB10*-OE fruits. Values are means ±SD of three biological replicates normalized using *FaACTIN* as an internal control. The Δ*C*T of EV-OE or EV-RNAi sample was chosen as the calibrator. Statistical significance was determined by Student’s *t*-test: *P<0.05, **P<0.01.

We also examined the expression of ripening-related genes in *FaMYB44.2*-OE and *FaMYB44.2*-RNAi fruits (Fig. 4A). We found that many structural genes were affected by FaMYB44.2, especially genes involved in sugar and acid metabolism (Fig. 4A). *FaSUS1* (*gene12940*), *FaSPS1*–*3* (*gene11606*/*22863*/*31122*) and *FaSUT1* are critical genes for sucrose metabolism in strawberry fruits (Jia *et al.*, 2013; Vallarino *et al.*, 2015; Supplementary Table S7). *FaSPS1*–*3* are mainly involved in sucrose accumulation, and overexpressed *FaSPS3* could significantly increase sucrose content in strawberry fruits (Supplementary Fig. S1). *FaSUS1* is thought to function in the breakdown of sucrose into monosaccharides, and its expression is negatively correlated with sucrose content (Tian *et al.*, 2012; Zhao *et al.*, 2016*a*; Vallarino *et al.*, 2015). *FaSUT1* is demonstrated to be a positive regulator in sucrose accumulation, and overexpressed and silenced *FaSUT1* increased and decreased sucrose content, respectively, in strawberry fruits (Jia *et al.*, 2013). As shown in Fig. 4A and Supplementary Fig. S2, *FaSPS3* was significantly suppressed in *FaMYB44.2*-OE fruits and significantly up-regulated in *FaMYB44.2*-RNAi, which is consistent with the altered sucrose contents in *FaMYB44.2*-OE and *FaMYB44.2*-RNAi fruits, suggesting that FaMYB44.2 might regulate sucrose accumulation by suppressing the expression of *FaSPS3*. *FaSUT1* was down-regulated in *FaMYB44.2*-OE fruits, which is in agreement with their reduced sucrose contents. The expression of other genes related to sugar accumulation, such as *FaHXK2* and *FaTPS7*, was also affected by FaMYB44.2 to different degrees (Fig. 4A). The specific role of *FaHXK2* and *FaTPS7* in sucrose metabolism needs to be further revealed.

We monitored the changes in expression of important genes encoding regulatory proteins associated with fruit development and ripening, such as *FaMRLK47*, *FaSnRK2.6*, *FaMYB1*, *FaMYB10*, and *FaMADS9*, as well as hormone-related genes (Aharoni *et al.*, 2001; Seymour *et al.*, 2011; Medina-Puche *et al.*, 2014; Lin-Wang *et al.*, 2014; Feng, 2015; Han *et al.*, 2015; Kadomura-Ishikawa *et al.*, 2015; Jia *et al.*, 2017; Fig. 4B). The expression of genes encoding other important transcription factors, including *FaMADS9*, *FaMYB10*, *FaGAMYB*, and *FaMYB1*, was affected to varying degrees by transient overexpression or silencing of *FaMYB44.2*, suggesting that FaMYB44.2 plays diverse roles in regulating fruit ripening (Fig. 4B; Supplementary Fig. S3A). While *FaMADS9* was the most affected gene, we also detected the expression of *FaMYB44* and *FaMYB10* in *FaMADS9*-OE fruits, but no obvious changes of these genes were detected (Supplementary Fig. S3B). Notably, although FaMYB10 and FaMRLK47 had opposite effects on anthocyanin accumulation, *FaMYB10* and *FaMRLK47* were both down-regulated in *FaMYB44.2*-OE fruits. This consistent expression pattern between *FaMYB10* and *FaMRLK47* was also demonstrated by Jia *et al.* (2017), further implying that FaMYB10 functions in sucrose accumulation (Fig. 4B). We also investigated the expression of genes encoding candidate partners of FaMYB44.2 in MBW complexes. *FabHLH3* and *FaTTG1* expression was altered in *FaMYB44.2* transgenic fruits, whereas no obvious changes in *FabHLH33* expression were detected. Since *FabHLH33* is expressed at very low levels in strawberry fruits (see Supplementary Table S6), FaHLH3 and FaTTG1 are more likely to be partners of FaMYB44.2 in the formation of functional MBW complexes in strawberry fruits (Fig. 4B). Additionally, transient manipulation of *FaMYB44.2* expression affected the expression of hormone-related genes, including *FaPYL1*, *FaJAZ1*, *FaARF6B*, and *FaPIN8* (Fig. 4B). Taken together, these results show that FaMYB44.2 is widely involved in regulating genes involved in fruit development and ripening and genes related to hormone biosynthesis and signal transduction, suggesting that FaMYB44.2 has diverse biological functions beyond directly modulating the expression of structural genes (Fig. 4B).

We also examined whether the expression of sugar-related genes was altered in *FaMYB10*-OE fruits, and found that *FaSPS1* was induced by *FaMYB10* overexpression, but that neither *FaSUS1/2* nor *FaSPS3* expression was affected (Fig. 4C). Previous studies showed miRNA156 plays important roles in the regulation of sucrose signaling and *MYB10* expression (Yang *et al.*, 2013; Qian *et al.*, 2017); miRNA319 and miRNA399 were involved in sucrose signaling (Liu and Carroll, 2010; Ren and Tang, 2012); and miRNA159 regulates *FaGAMYB* (Csukasi *et al.*, 2012). To examine whether the regulation of FaMYB44.2 is related to miRNA, we determined the expression of these miRNAs, and the results indicated that miRNA159, miRNA156, and miRNA319 were changed in *FaMYB44.2* transgenic fruits, suggesting that FaMYB44.2 may have potential roles in miRNA regulation (see Supplementary Fig. S3C).

### FaMYB44.2 interacts with important transcription factors involved in regulating fruit ripening

**Fig. 5.**
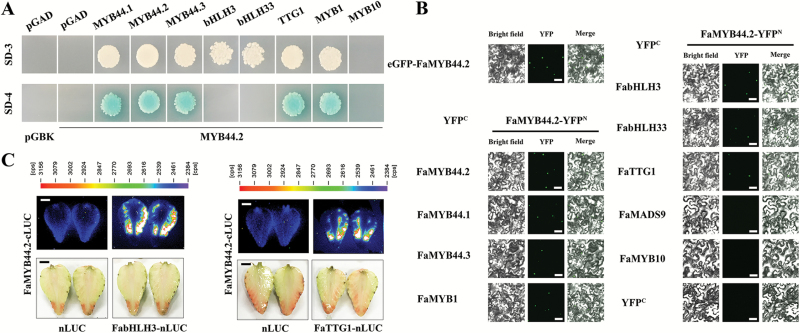
Protein interaction assay of FaMYB44.2. (A) Analysis of FaMYB44.2-interacting proteins via yeast two-hybrid assay. (B) Protein interactions and subcellular localization of FaMYB44.2. The interactions were examined by BiFC assay. Scale bar: 20 µm. (C) Firefly luciferase complementation imaging assay of FaMYB44.2 in strawberry fruits. Scale bar: 0.5 cm.

As shown in Fig. 1, FaMYB44.2 is an R2R3 transcription factor containing a transcription repressor-related region. To verify the transcriptional activity of FaMYB44.2, we co-transformed yeast cells with pGADT7 and pFaMYB44.2-BK. The transformed cells failed to grow on either SD−Trp/−Leu/−His (SD-3) or SD−Trp/−Leu/−His/−Ade (SD-4) medium, indicating that FaMYB44.2 by itself does not promote downstream gene expression in yeast (Fig. 5A; Supplementary Fig. S4).

We further investigated the interactions of FaMYB44.2 with potential partners in the MBW complex by Y2H and bimolecular fluorescence complementation (BiFC). BiFC analysis showed that FabHLH3, FabHLH33, and FaTTG1 physically interact with FaMYB44.2 in tobacco (*Nicotiana tabacum*) leaves (Fig. 5B). However, yeast cells co-transformed with FabHLH3-AD/FaMYB44.2-BK or FabHLH33-AD/FaMYB44.2-BK failed to grow on SD-4 medium (Fig. 5A; Supplementary Fig. S4). An LCI assay showed FaMYB44.2 interacted with FabHLH3 and FaTTG1 in strawberry fruits, suggesting they may be subunits of FaMYB44.2-related MBW complexes (Fig. 5C). However, whether FabHLH3, FabHLH33, and FaTTG1 help FaMYB44.2 regulate downstream gene expression in fruits, and the relationship between FaTTG1, FabHLH3, FabHLH33, and sucrose accumulation merit further exploration. We also found that FaMYB44.2 interacted with itself and with two other S22 members, FaMYB44.1 and FaMYB44.3, indicating that FaMYB44.2 can form homodimers and heterodimers (Fig. 5A, B). Indeed, AtMYB44 can also form homodimers (Persak *et al.*, 2013), but the functions of any possible FaMYB44.2 homodimers and heterodimers remain unknown.

Manipulating *FaMYB44.2* expression affected the expression of several important regulatory genes (Fig. 4B). However, whether FaMYB44.2 also affects their functions through direct physical interactions is unknown. Therefore, we investigated the interactions between FaMYB44.2 and other important regulatory proteins involved in strawberry fruit ripening, such as FaMYB1, FaMYB10, and FaMADS9. In both yeast cells and tobacco leaves, FaMYB44.2 interacted with FaMYB1, but not with FaMYB10 or FaMADS9 (Fig. 5A, B).

### FaMYB44.2 physically interacts with elements in the *FaSPS3*, *FaSUS1*, *FaHXK2*, *FaMDH1*, and *FaCS1* promoters

**Fig. 6.**
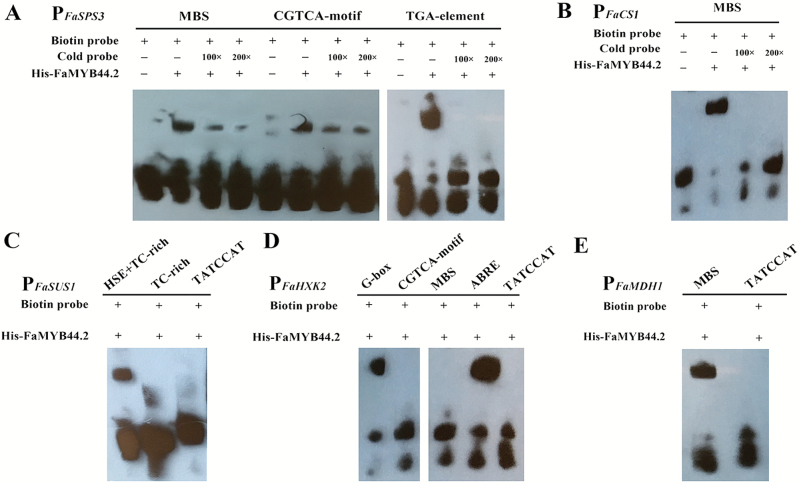
Validating the binding of structural genes directly downstream of FaMYB44.2 by EMSA. (A) FaMYB44.2 physically interacts with the *FaSPS3* promoter (P_*FaSPS3*_). The binding of MBS and CGTCA motif was detected in one experiment while the competitive binding of TGA element was performed separately. (B) EMSA between FaMYB44.2 and the *FaCS1* promoter (P_*FaCS1*_). The specific binding between FaMYB44.2 and P_*FaCS1*_ was validated using different concentrations of cold competitive probes. (C) EMSA showing the interaction of FaMYB44.2 with the *FaSUS1* promoter. (D) EMSA showing the interaction of FaMYB44.2 with the *FaHXK2* promoter; the binding of G-box and CGTCA motif was detected in one experiment while the binding of MBS, ABRE and TATCCAT elements was performed in another experiment. (E) EMSA showing the interaction of FaMYB44.2 with the *FaMDH1* promoter. ABRE, *cis*-acting element involved in the ABA response; CGTCA motif, *cis*-acting regulatory element involved in the MeJA response; G-box, cis-acting regulatory element involved in the light response; HSE, *cis*-acting element involved in the heat stress response; MBS, MYB binding site involved in drought inducibility; TATCCAT, *cis*-acting element involved in repressing the sugar response with a G-box; TC-rich, *cis*-acting element involved in defense and stress responses; TGA, auxin-responsive element.

We demonstrated that the expression levels of several important structural genes involved in sugar and organic acid metabolism were significantly altered (Fig. 4A). We therefore investigated the interactions between FaMYB44.2 and the *cis*-regulatory elements in the promoters of *FaSPS3*, *FaSUS1*, *FaHXK2*, *FaMDH1*, and *FaCS1*, whose expression was significantly altered in response to transiently expressed *FaMYB44.2*. Since sucrose and acid content can be affected by hormonal and environmental factors, in addition to MYB-binding sites (MBS), we also examined the interactions between FaMYB44.2 and elements in the promoters of genes responsive to hormonal and environmental factors, including ABA, JA, and temperature. As shown in Fig. 6, FaMYB44.2 interacted with the MBS elements in *FaSPS3*, *FaCS1*, and *FaMDH1*, the ABRE and G-box elements in *FaHXK2*, and the TC-rich + HSE regions of *FaSUS1*. Although the promoters of *FaSPS3*, *FaSUS1*, *FaMDH1*, *FaCS1*, and *FaHXK2* all contain FaMYB44.2-binding elements, the number and type of binding element differ among genes. Therefore, although these genes might function as structural genes downstream of FaMYB44.2, their regulatory mechanisms are not the same, and each structural gene might couple with FaMYB44.2 to participate in diverse signaling pathways controlled by various upstream signals. Furthermore, as shown in Fig. 6A, FaMYB44.2 interacted with three different elements in the promoter of *FaSPS3* (P_*FaSPS3*_), i.e. MBS, JA-responsive element, and auxin-responsive element. Therefore, based on the key role of *FaSPS3* in sucrose accumulation in strawberry fruits (Vallarino *et al.*, 2015; Supplementary Fig. S1), we chose *FaSPS3* as the major downstream gene of FaMYB44.2 to further uncover the mechanisms of FaMYB44.2-modulated sucrose accumulation.

### FaMYB44.2 modulates sucrose accumulation by repressing the activity of P_*FaSPS3*,_ which is enhanced by MeJA in strawberry fruits

**Fig. 7.**
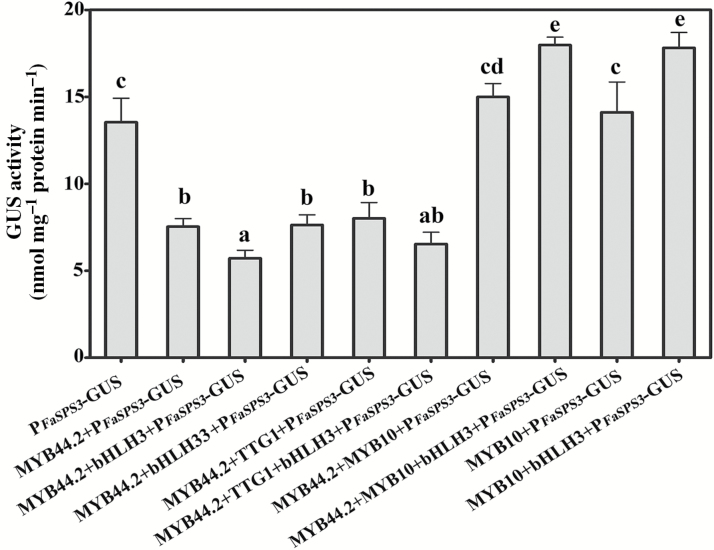
P_*FaSPS3*_ activity assay in strawberry fruits. GUS activity was used to detect the activity of P_*FaSPS3*_. *FaMYB10*, *FaMYB44.2*, *FabHLH*, and *FaTTG1* were used as the reporter genes. Three biological replicates were performed. Statistically significant differences among samples were tested by Turkey’s test, and significant differences at the *P*<0.05 level are indicated by different letters.

**Fig. 8.**
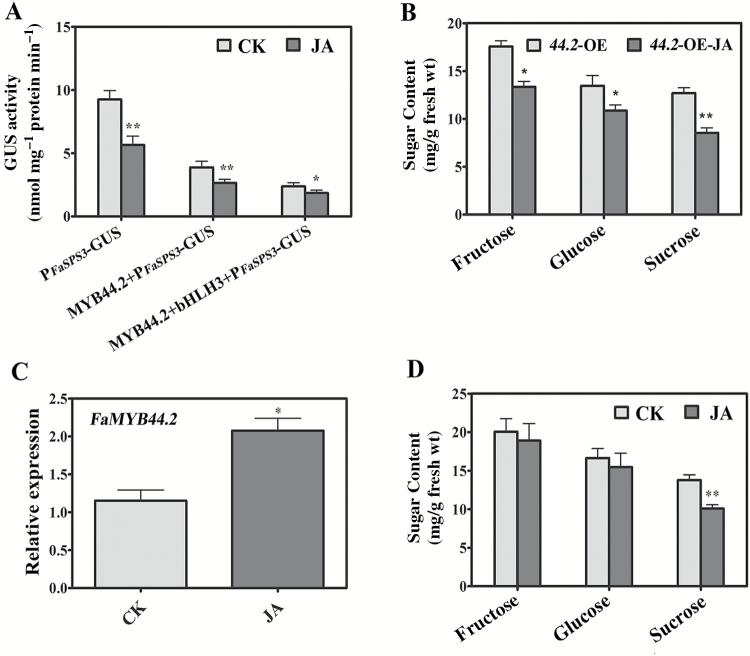
JA enhances FaMYB44.2-repressed sucrose accumulation in strawberry fruit. (A) MeJA enhances the repressive activity of FaMYB44.2 on P_*FaSPS3*_ in strawberry fruits. (B) Sugar contents in *FaMYB44.2*-OE strawberry fruits decreased in response to spraying with 100 µM MeJA. (C) MeJA induces the expression of *FaMYB44.2*. Values are means ±SD of three biological replicates. (D) MeJA reduces sucrose accumulation in strawberry fruits. Statistically significant differences from control were determined by Student’s *t*-test: *P<0.05; **P<0.01.

To verify the role of FaMYB44.2 in *FaSPS3*-mediated sucrose accumulation, we used GUS driven by the *FaSPS3* promoter (P_*FaSPS3*_) as the reporter and FaMYB44.2 as the effector in a *trans*-activation assay to clarify the regulatory mechanisms of FaMYB44.2-modulated sucrose accumulation in strawberry fruits. Compared with fruits transformed with P_*FaSPS3*_::GUS, GUS activity was lower in fruits transformed with FaMYB44.2-P_*FaSPS3*_::GUS. FabHLH3 enhanced this repression (Figs 7, 8A), suggesting that FaMYB44.2 regulates sucrose accumulation by repressing the expression of *FaSPS3* in strawberry fruits and that FabHLH3 functions as a co-repressor in this negative regulatory process.

As shown in Fig. 6A, FaMYB44.2 interacted with the *FaSPS3* promoter by interacting with its CGTTCA motif, which is involved in JA responses. We therefore examined the effects of MeJA on the ‘FaMYB44.2-*FaSPS3*’ module, and found that the repressive effect of FaMYB44.2 and FabHLH3 on P_*FaSPS3*_ activity was enhanced by MeJA (Fig. 8A). Notably, MeJA further enhanced the decrease in main sugar contents in *FaMYB44.2*-OE fruits (Fig. 8B) and MeJA induced the expression of *FaMYB44.2* (Fig. 8C). These results suggest that JA might be involved in sucrose accumulation in strawberry fruit and that FaMYB44.2 might function as its downstream component. To investigate whether JA regulates sucrose accumulation in strawberry fruit, we treated detached strawberry fruits with 100 µM MeJA. Compared with control fruits, MeJA reduced sucrose accumulation in strawberry fruits but had no obvious effect on glucose or fructose levels (Fig. 8D), suggesting that JA-repressed sugar accumulation is dependent on *FaMYB44.2* expression.

### The repressive effect of FaMYB44.2 on *FaSPS3* is reversed by FaMYB10 in strawberry fruits

**Fig. 9.**
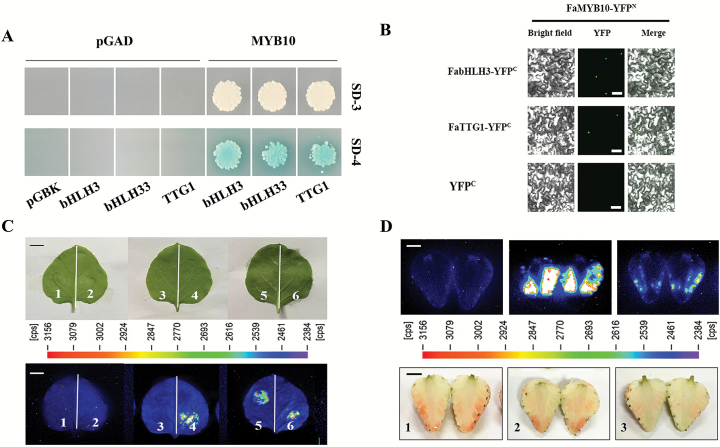
Effects of FaMYB10 on the interaction of FaMYB44.2 with FabHLH3 or FaTTG1. (A) Detection of FaMYB10-interacting proteins by Y2H. (B) Detection of FaMYB10-interacting proteins by BiFC. Scale bar: 20 µm. (C) Effects of FaMYB10 on the interaction between FaMYB44.2 and FabHLH3 in tobacco leaves. 1, co-transformation of nLUC and cLUC; 2, co-transformation of nLUC and cLUC-FaMYB44.2; 3, co-transformation of FaMYB44.2-nLUC and cLUC; 4, co-transformation of FaMYB44.2-nLUC, cLUC-FabHLH3 and pBI121; 5, co-transformation of FaMYB44.2-nLUC, cLUC-FabHLH3, and pBI121-FaMYB10 (1:1:1); 6, co-transformation of FaMYB44.2-nLUC, cLUC-FabHLH3, and pBI121-FaMYB10 (1:1:5). Scale bar: 0.5 cm. (D) Effects of FaMYB10 on the interaction between FaMYB44.2 and FabHLH3 in strawberry fruits. 1, co-transformation of FaMYB44.2-nLUC and cLUC; 2, co-transformation of FaMYB44.2-nLUC and cLUC-FabHLH3; 3, co-transformation of FaMYB44.2-nLUC, cLUC-FabHLH3, and pBI121-FaMYB10 (1:1:3). Scale bar: 0.5 cm.

As indicated above, FaMYB10 may be associated with sugar accumulation in strawberry fruits (Rolland *et al.*, 2006; Medina-Puche *et al*., 2014; Lin-Wang *et al.*, 2014; Fig. 4). To clarify the role of FaMYB10 in sugar metabolism and its relationship with FaMYB44.2-mediated sucrose accumulation, we co-transformed fruits with *FaMYB10*, *FaMYB44.2*, and *FabHLH3* to examine the effects of FaMYB10 on sucrose accumulation, as modulated by the ‘FaMYB44.2–FabHLH3–FaSPS3’ module. The repression of FaMYB44.2 was reversed by FaMYB10, and FabHLH3 enhanced this effect, suggesting that when co-transformed into strawberry fruits, FaMYB10 competitively binds to FabHLH3 over FaMYB44.2 (Fig. 7). To verify whether FaMYB10 reverses GUS activity by regulating the expression of *FaSPS3* or via interplay with FaMYB44.2, we co-expressed FaMYB10 and P_*FaSPS3*_::GUS in strawberry fruits. FaMYB10 is unable to increase the activity of P_*FaSPS3*_ by itself, but when co-transformed with FabHLH3, this effect was significantly enhanced (Fig. 7). Analysis of gene expression in *FaMYB10*-OE showed that the expression of *FaSPS3* was not significantly affected (Fig. 4C), suggesting that FaMYB10 primarily regulates the expression of *FaSPS3* by reversing the repressive effect of FaMYB44.2 on *FaSPS3* or by coordinating with FabHLH3, rather than regulating the expression of *FaSPS3* by itself. We further investigated whether FaMYB10 interacts with FabHLH3 via Y2H and BiFC assays. As shown in Fig. 9A, B, FaMYB10 physically interacts with FabHLH3, FabHLH33, and FaTTG1. To further analyse whether FaMYB10 affects the interaction between FaMYB44.2 and FabHLH3, we co-transformed tobacco leaves and strawberry fruits with FaMYB10 and FaMYB44.2-cLUC/FabHLH3-nLUC. When co-transformed with FaMYB10, the interaction between FaMYB44.2 and FabHLH3 was reduced in both tobacco leaves and strawberry fruits (Fig. 9C, D). These results suggest that FaMYB10 regulates the functioning of FaMYB44.2 not only by repressing its expression (Fig. 4C), but also by suppressing the interaction of FaMYB44.2 with its co-repressor, FabHLH3 (Figs 7, 9C, D). We also detected the effects of FaMADS9 on FaMYB44.2/FaMYB10-modulated GUS activity; the results showed that FaMADS9 has no obvious effect on FaMYB44/FaMYB10-modulated *FaSPS3* function (see Supplementary Fig. S5).

## Discussion

**Fig. 10.**
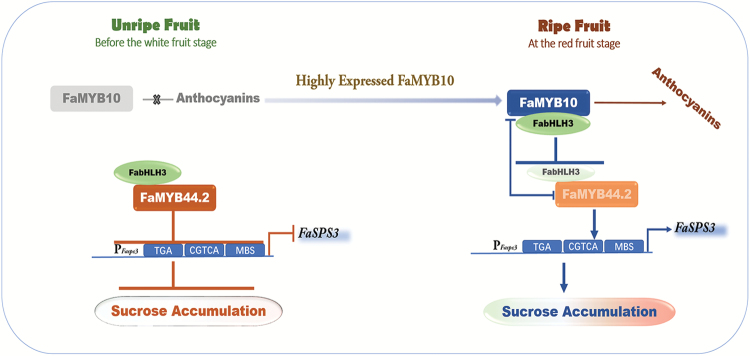
Hypothetical model of the role of FaMYB44.2 in the regulation of *FaSPS3* during fruit ripening. *FaSPS3* is the central structural gene involved in sucrose accumulation; its high level of expression contributes to ripening-related sucrose accumulation. In unripe fruit, FaMYB44.2 and its co-repressor FabHLH3 form a complex that represses the expression of *FaSPS3*. Due to the absence of FaMYB10, this repression is strong, and thus the expression of *FaSPS3* remains at low levels and the sucrose content is low. As fruit ripening progresses, at the white fruit stage, FaMYB10 is present in strawberry fruits and its expression remains at higher levels than FaMYB44.2 during the subsequent ripening stages. High levels of FaMYB10 reverse the repression of FaMYB44.2 and competitively bind to FabHLH3, a co-repressor of FaMYB44.2, to further induce the expression of *FaSPS3*; thus, the fruits tend to accumulate sucrose. In addition, FaMYB10 limits the expression of FaMYB44.2 and vice versa, thereby balancing ripening-related metabolism. Eventually, the antagonistic interplay between FaMYB10 and FaMYB44.2 leads to the accumulation of both sucrose and anthocyanins in ripe strawberry fruits. Arrows indicate activation and T-bars show repression. This model was constructed based on the results of the current study, as well as those of Vallarino *et al.* (2015) and Lin-Wang *et al.* (2014).

In this study, we identified the R2R3 MYB transcriptional repressor FaMYB44.2 and demonstrated that it regulates sucrose accumulation through repressing the expression of *FaSPS3* (Figs 3, 4, 6, 7). Furthermore, we showed that a positive regulator of anthocyanin biosynthesis, FaMYB10, is involved in this repressive regulation (Figs 4, 7, 9). Based on these findings, we constructed a model for the role of FaMYB44.2 as a negative regulator of sucrose accumulation in strawberry fruits (Fig. 10). According to this model, before the white fruit stage, the transcriptional repressor FaMYB44.2 inhibits sucrose accumulation with or without FabHLH3 by directly repressing the expression of key structural genes, such as *FaSPS3*. At the white fruit stage, FaMYB10 is expressed, and FaMYB10 and FaMYB44.2 are both expressed at high levels during the subsequent ripening stages, whereas their antagonistic expression patterns prevent excess increases in FaMYB10 or FaMYB44.2 levels. Meanwhile, higher levels of FaMYB10 resulted in its competitive advantage in binding to FabHLH3 (Figs 7, 9; Supplementary Table S8). Collectively, our results indicate that the role of the ‘FaMYB10–FaMYB44.2–FabHLH3’ module in regulating *FaSPS3*-mediated sucrose accumulation is achieved via the following steps: (i) higher expression of FaMYB10 reverses the repressive effect of FaMYB44.2 on *FaSPS3* (Figs 2, 7; Supplementary Table S9); (ii) FaMYB10 competitively binds to FabHLH3 to weaken the repressive effect of FaMYB44.2 on *FaSPS3* (Figs 7, 9D); and (iii) FaMYB10 cooperates with FabHLH3 to activate the expression of *FaSPS3* (Fig. 7). The antagonistic interplay between FaMYB10 and FaMYB44.2 ultimately results in the accumulation of sucrose in ripening strawberry fruits, and the relative expression level between FaMYB10 and FaMYB44 may act as the molecular switch to regulate sucrose accumulation in strawberry fruits.

In a recent study, StMYB44 was found to negatively regulate P_i_ transport in potato by suppressing the expression of *StPHO1*. Stable transgenic *StMYB44*-OE plants and tubers were significantly smaller than the controls, suggesting that StMYB44 affects metabolism in plants and tubers. RNA-sequence analysis showed that sugar metabolism-related genes such as beta-fructofuranosidase and sugar transporter genes were up-regulated in *StMYB44*-OE versus the control (Zhou *et al.*, 2017). Moreover, StMYB44 may function via redundant mechanisms, as no obvious phenotypes were observed in CRISPR/Cas9-mediated *StMYB44* knockout plants (Zhou *et al.*, 2017). In the current study, we noticed that FaMYB44.2 formed homodimers and heterodimers in plants, such as AtMYB44 in Arabidopsis (Persak *et al.*, 2013; Fig. 5A, B). However, the true functions of other FaMYB44 proteins and the biological significance of these homodimers and heterodimers, as well as whether redundant mechanisms exist among FaMYB44.1/44.2/44.3, remain to be determined.

FaMYB10 is a major regulatory protein that promotes anthocyanin biosynthesis in the Rosaceae (Medina-Puche *et al.*, 2014; Lin-Wang *et al.*, 2014). Although previous studies have suggested that FaMYB10 is associated with the expression of sucrose-related genes, the role of MYB10 in sugar accumulation has not previously been reported (Medina-Puche *et al.*, 2014). In the current study, we showed that the expression of *FaSPS1* was changed in *FaMYB10*-OE fruits (Fig. 4C) and that the interplay between FaMYB44.2 and FaMYB10 resulted in the accumulation of sucrose in strawberry fruits (Figs 4B, 7, 9). However, several issues need to be addressed to clarify the functions of FaMYB10 in sucrose accumulation. Although we found that FaMYB10 and FaMYB44.2 did not directly interact (Fig. 5A, B), it remains unknown whether FaMYB10 directly regulates the expression of *FaMYB44.2* and if it directly regulates the expression of *FaSPS1/2/3* via directly binding to and activating the promoters of these genes. Furthermore, as shown in Fig. 4B, C, *FaMYB44.2* expression was suppressed by the overexpression of *FaMYB10* and vice versa, suggesting that a balance needs to be maintained between FaMYB44.2 and FaMYB10 to prevent overactivation of a single metabolic pathway, thereby allowing the fruit to develop a rich flavor. This balance might not only function in sucrose accumulation. Nevertheless, whether FaMYB44.2 also affects FaMYB10-modulated anthocyanin levels remains unknown. In Arabidopsis, MYB44 plays multiple roles in regulating plant growth and stress/disease resistance (Jung *et al.*, 2008; Jaradat *et al.*, 2013; Shim *et al.*, 2013; Persak and Pitzschke, 2014). The overexpression of *MYB44* increased disease resistance in plants, but also caused abnormal growth, including reduced plant size and delayed flowering (Jung *et al.*, 2008; Zhou *et al.*, 2017). The interplay between FaMYB10 and FaMYB44.2, whose levels are balanced by mutual inhibition, may play important roles in regulating fruit ripening and the responses to stress and diseases.

We also found that FaMYB44.2 regulates many regulatory and key hormone-related genes (Fig. 4B), which is in agreement with the multiple roles of MYB44 in Arabidopsis. Comprehensive analysis of our results and those of studies in other species suggested that FaMYB44.2 not only functions downstream during sucrose accumulation by directly binding to structural genes, but also functions as an upstream signaling component that modulates other biological processes. To fully clarify the roles of FaMYB44.2, stable transgenic FaMYB44.2 plants should be created via overexpression or CRISPR, but when constructing knockout mutants, the redundant mechanisms involving MYB44 need to be taken into consideration (Zhou *et al.*, 2017).

To date, little is known about the regulatory mechanisms of sucrose accumulation in strawberry fruits. We previously demonstrated that the protein kinase FaMRLK47 is a positive regulator of sucrose accumulation, and Vallarino *et al.* found that in *FaGAMYB*-silenced strawberry receptacles, sucrose accumulation and *FaSPS1–3* expression were reduced (Vallarino *et al.*, 2015; Jia *et al.*, 2017). Similarly, in the current study, we found that *FaSPS1–3* and sucrose accumulation were down-regulated in *FaMYB44.2*-OE fruits and up-regulated in *FaMYB44.2*-RNAi fruits (Fig. 4A). However, for *FaSUS1/2*, an opposite expression pattern was detected in *FaGAMYB*-silenced and *FaMYB44.2*-OE fruits (Fig. 4A), suggesting that FaGAMYB and FaMYB44.2 play different regulatory roles in sucrose breakdown (Vallarino *et al.*, 2015).

The quality of strawberry fruit is affected by acid and sugar levels and their ratios (Montero *et al.*, 1996; Basson *et al.*, 2010; Concha *et al.*, 2013; Gündüz and Özdemir, 2014). We found that FaMYB44.2 regulates acid content in fruit (Figs 3C, 4A, 6B, E; Table 1), but the underlying mechanisms are complex. We also used a reporter and effector assay to analyse the role of the FaMYB44.2-mediated pathway in acid accumulation. However, we found that although the expression of downstream structural genes was reduced, the GUS activity driven by the promoters of these structural genes was not significantly regulated by FaMYB44.2, at least for *FaCS1* and *FaMDH1*; therefore, the roles of FaMYB44.2 in acid metabolism need to be further analysed.

MeJA can improve the sugar/acid ratio in strawberry fruits (Concha *et al.*, 2013). Indeed, we found that MeJA repressed the accumulation of sucrose in strawberry fruit (Fig. 7E), which is consistent with the effects of MeJA on sucrose accumulation in strawberry fruits previously described (Giné-Bordonaba and Terry, 2016). The effects of MeJA on sugar metabolism differ in different cultivars and for different sugar components (Pérez *et al.*, 1997; Giné-Bordonaba and Terry, 2016). Due to the complex roles and the concentration-dependent property of JA, the effects of JA on sugar accumulation should be further explored. We also found that MeJA was involved in FaMYB44.2–*FaSPS3*-modulated sucrose accumulation (Fig. 8B, C, D) and that the expression of JA biosynthesis and signaling transduction genes such as *FaOPR3* and *FaJAZ1* was changed by FaMYB44.2 (Fig. 4B), suggesting that the function of FaMYB44.2 is highly related to MeJA.

Taken together, we identified a novel transcriptional repressor, FaMYB44.2, involved in sugar and acid accumulation in strawberry fruits. Further analysis showed that the regulatory role of FaMYB44.2 in sucrose accumulation is associated with FaMYB10 and MeJA and that the interplay between FaMYB10 and FaMYB44.2 ultimately results in sucrose accumulation in ripe strawberry fruits. This study provides new clues about the regulatory mechanisms of sucrose accumulation, laying the foundation for further investigations of the interplay among regulatory proteins during strawberry fruit ripening and quality formation.

## Supplementary data

Supplementary data are available at *JXB* online.

Additional supporting information may be found in the online version of this article.

Fig. S1. The effects of *FaSPS3*-OE on sucrose accumulation in strawberry fruits.

Fig. S2. Detection of the expression levels of *FaACTIN*, *FaSUS*, and *FaSPS3* in strawberry fruits.

Fig. S3. The expression levels of *miRNA* and *MADS* in transgenic *FaMYB44.2* and *FaMADS9* fruits.

Fig. S4. Identification of the interaction proteins of FaMYB44.2 and FaMYB10 by yeast two-hybrid assay.

Fig. S5. The effects of FaMDS9 on FaMYB44.2/FaMYB10-mediated P_*FaSPS3*_ activity.

Table S1. The full-length protein sequences for *MYB44* and *MYB1* genes.

Table S2. Primers used for vector construction.

Table S3. Primers used for gene expression analysis by qRT-PCR.

Table S4. Primers used to construct the vectors for the GUS activity assay.

Table S5. Probes used for the EMSA.

Table S6. Sequences and primers used for microRNA expression analysis by qRT-PCR.

Table S7. Primers used for gene expression analysis by RT-PCR.

Table S8. *FaSUS*, *FaSPS*, and *FabHLH33* expression at different developmental and ripening stages according to RNA-sequence data.

Table S9. Quantitative analysis of *FaMYB10* and *FaMYB44.2* expression levels by qRT-PCR.

## Supplementary Material

Supplementary Figures S1-S5 and Tables S2-S9

Supplementary Table S1
