# Supplementary material for: FaMYB44.2, a transcriptional repressor, negatively regulates sucrose accumulation in strawberry receptacles through interplay with FaMYB10
Source: J Exp Bot. 2018 Aug 2;69(20):4805–20. doi: 10.1093/jxb/ery249 (PMC6137983; doi:10.1093/jxb/ery249)
Supplement: Supplementary Figures S1-S5 and Tables S2-S9 [file ery249_suppl_supplementary_figures_s1-s5_tables_s2-s9.pdf]

Supplementary Data

FaMYB44.2 negatively regulates sucrose accumulation in strawberry receptacles through interplay with FaMYB10

Lingzhi Wei, Wenwen Mao, Meiru Jia, Sinian Xing, Usman Ali, Yaoyao Zhao, Yating Chen, Minglin Cao, Zhengrong Dai, Kai Zhang, Zhechao Dou, Wensuo Jia and Bingbing Li

Figures and Tables

Fig. S1. The effects of *FaSPS3*-OE on sucrose accumulation in strawberry fruits.

Fig. S2. Detect the expression levels of *FaACTIN*, *FaSUS* and *FaSPS3* in strawberry fruits.

Fig. S3. The expression levels of *miRNA* and *MADS* in transgenic *FaMYB44.2* and *FaMADS9* fruits.

Fig. S4. Identify the interaction proteins of FaMYB44.2 and FaMYB10 by yeast two-hybrid assay.

Fig. S5. The effects of FaMDS9 on FaMYB44.2/FaMYB10-mediated P_{FaSPS3} activity.

Table S1. The full-length protein sequences for MYB44 and MYB1 genes.

Table S2. Primers used for vector construction.

Table S3. Primers used for gene expression analysis by qRT-PCR.

Table S4. Primers used to construct the vectors for the GUS activity assay.

Table S5. Probes used for the EMSA.

Table S6. Sequences and primers used for microRNA expression analysis by qRT-PCR.

Table S7. Primers used for gene expression analysis by RT-PCR.

Table S8. *FaSUS*, *FaSPS and FabHLH33* expression at different developmental and ripening stages according to RNA-sequence data.

Table S9. Quantitative analysis of *FaMYB10* and *FaMYB44.2* expression levels by qRT-PCR.

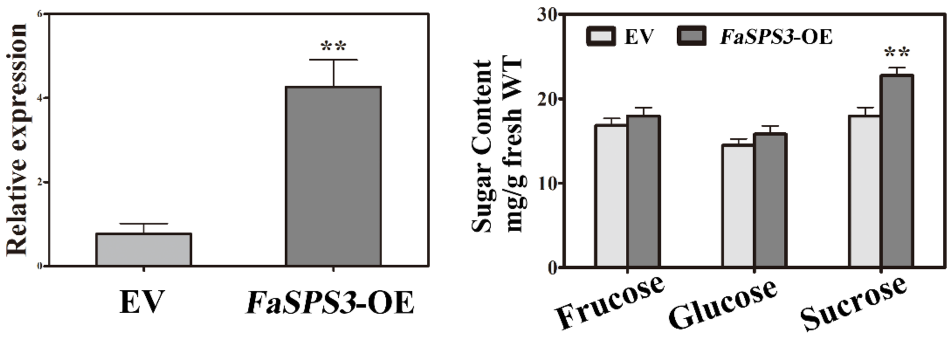

Supplementary Figure S1. The effects of *FaSPS3*-OE on sucrose accumulation in strawberry fruits. *FaSPS3* was overexpressed in white strawberry fruits using pBI121 vector, and the main sugar contents were analyzed by HPLC assay. Values are means ± SD of three biological replicates and normalized using *FaACTIN* as an internal control. The ΔCT of EV sample was chosen as the calibrator. Statistically significant were tested by Student’s t test: *P < 0.05 and **P < 0.01. Thirty fruits were pooled as one sample and three biological replicates were performed.

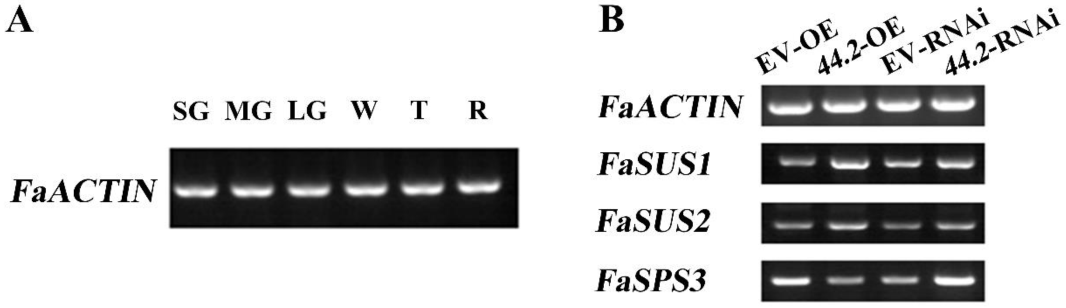

Supplementary Figure S2. Detect the expression levels of *FaACTIN, FaSUS* and *FaSPS3* in strawberry fruits. A, The expression pattern of *FaACTIN* during strawberry fruit ripening. SG, small green fruit; MG, middle green fruit; LG, large green fruit; W, white fruit; T, turning fruit and R, red fruit. **B,** The expression levels of *FaSUS1*, *FaSUS2* and *FaSPS3* in *FaMYB44.2*-OE and *FaMYB44.2*-RNAi fruits.

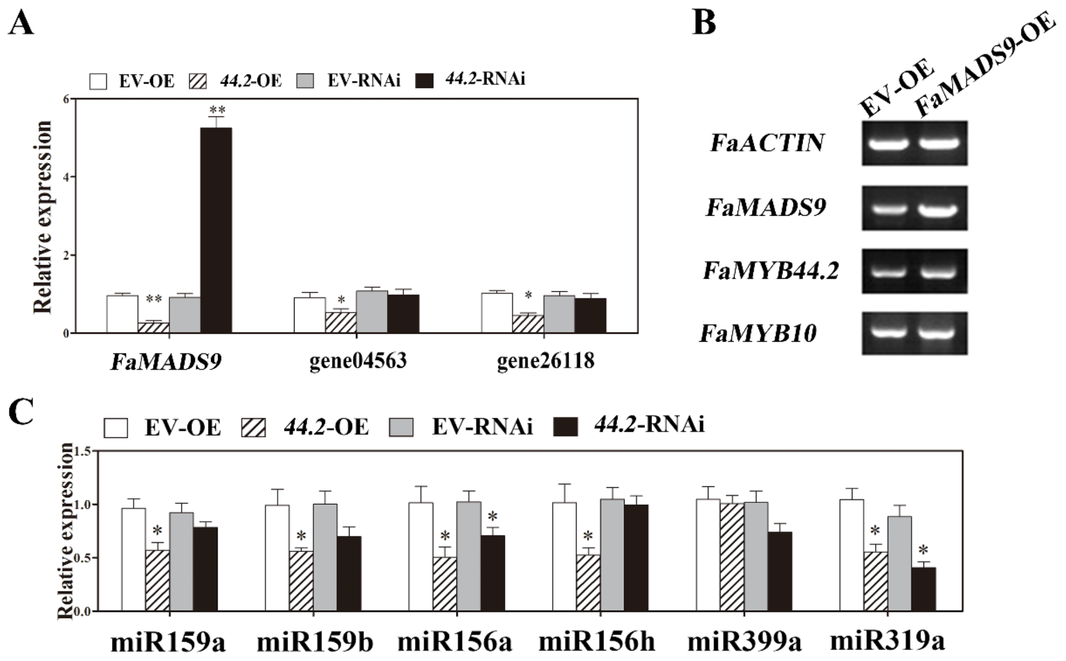

Supplementary Figure S3. The expression levels of *miRNA* and *MADS* in transgenic *FaMYB44.2* and *FaMADS9* fruits. A, The expression levels of *FaMADS9* and its homologues in *FaMYB44.2*-OE and *FaMYB44.2*-RNAi fruits (Feng, 2015). **B**, *FaMADS9* was overexpressed in white strawberry fruits using pSPYCE vector, and the expression of *FaMADS9*, *FaMYB44.2* and *FaMYB10* was analyzed with RT-PCR. **C**, The expression levels of *miRNA 159*, *miRNA156*, *miRNA399* and *miRNA319* (Liu and Carroll, 2010; Csukasi *et al.*, 2012; Ren and Tang, 2012; Yang *et al.*, 2013; Qian *et al.*, 2017) in *FaMYB44.2*-OE and *FaMYB44.2*-RNAi fruits. The primers for RT-PCR were listed in Table S7.

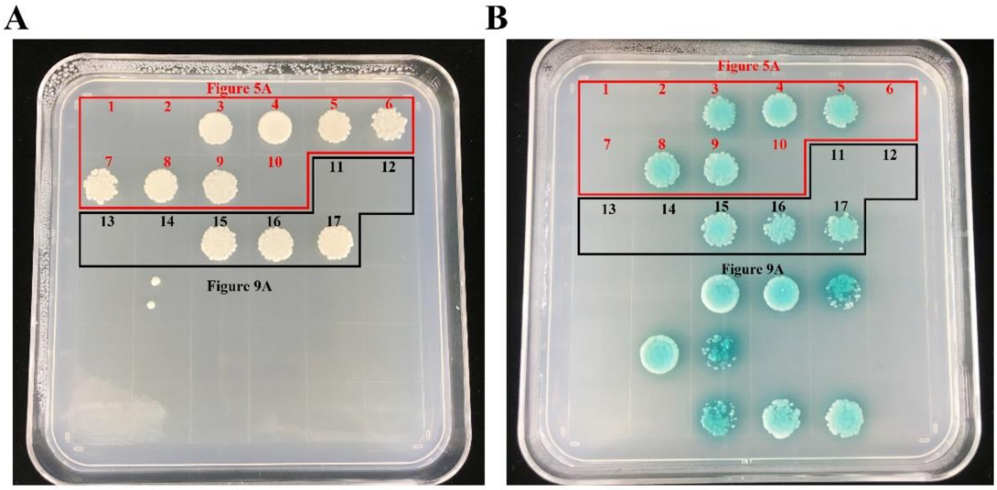

Supplementary Figure S4. Identify the interaction proteins of FaMYB44.2 and FaMYB10 by yeast two-hybrid assay. A, –Leu/–Trp/–His (SD-3) of Fig. 5A. 1, pGAD+pGBK; 2, pGAD+BD-FaMYB44.2; 3, AD-FaMYB44.1+BD-FaMYB44.2; 4, AD-FaMYB44.2+BD-FaMYB44.2; 5, AD-FaMYB44.3+BD-FaMYB44.2; 6, AD-FabHLH3+BD-FaMYB44.2; 7, AD-FabHLH33+BD-FaMYB44.2; 8, AD-FaTTG1+BD-FaMYB44.2; 9, AD-FaMYB1+BD-FaMYB44.2; 10, AD-FaMYB10+BD-FaMYB44.2. –Leu/–Trp/–His (SD-3) of Fig. 9A. 11, pGAD+pGBK; 12, pGAD+BD-FabHLH3; 13, pGAD+BD-FabHLH33; 14, pGAD+BD-FaTTG1; 15, AD-FaMYB10+BD-FabHLH3; 16, AD-FaMYB10+BD-FabHLH33; 17, AD-FaMYB10+BD-FaTTG1. **B,** –Leu/–Trp/–His/–adenine (SD-4) of Fig. 5A and Fig. 9A. The panel was in the same order as shown in Fig. S4A.

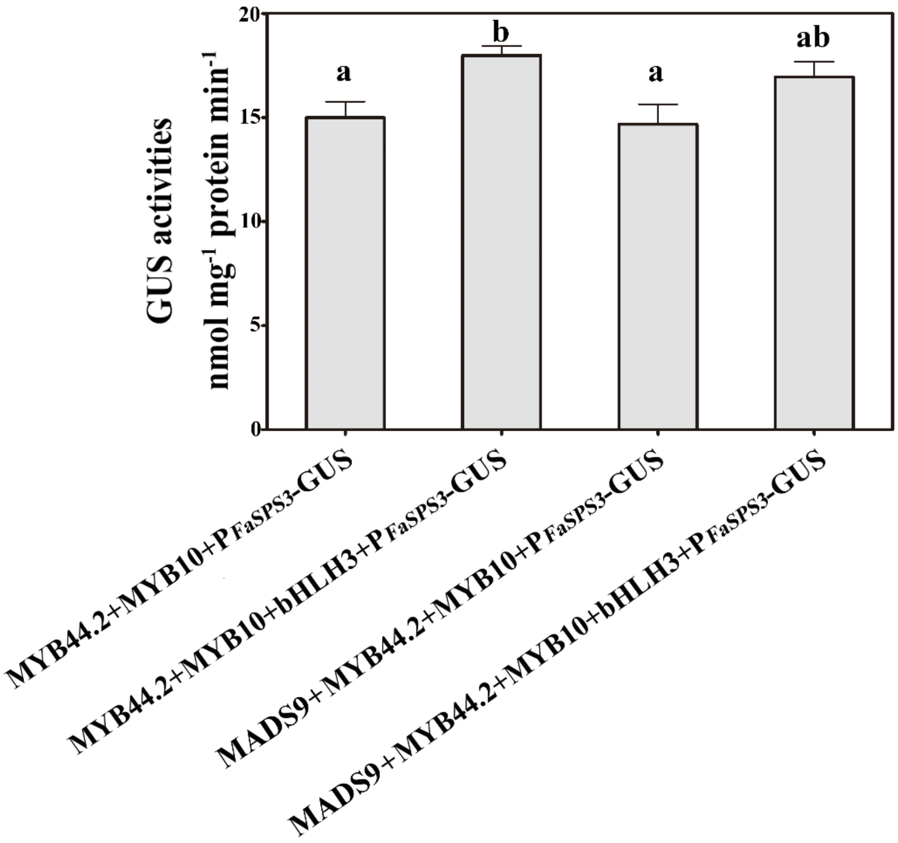

Supplementary Figure S5. Detecting the effects of FaMDS9 on FaMYB44.2/FaMYB10-mediated P*FaSPS3* activity.

Table S1. The full-length protein sequences for MYB44 and MYB1 genes. Please see the details in the attached Excel file.

Table S2. Primers used for vector construction. The restriction enzyme sites in the primer sequences are underlined.

Primer name	Enzyme sites	Forward primer (5'-3')	Reverse primer (5'-3')
***FaMYB44.2*-OE**	Gateway adapters	AAAAAGCAGGCTATGGCTTCTTCTTCAACCAAGA	AGAAAGCTGGGTCTACTCCACCTTGCTAATCCCAA
***FaMYB44.2*-RNAi-P**	AscI/SwaI	GGCGCGCCATCACCCCTCTTCTCTCTCAGAACAA	ATTTAAATTACGCCGGGCAGACTAGAATCGCTC
***FaMYB44.2*-RNAi-N**	PacI/AvrII	TTAATTAATCACCCCTCTTCTCTCTCAGAACAAG	CCTAGGGTACGCCGGGCAGACTAGAATCGC
***FaSPS3*-OE**	BamHI/SacI	GGATCCATGCCGGAGGGTACCAGAAC	GAGCTCCTAGTTCTTGACAACCTCTAATTTCTC
**pGEX-4T-1-MYB44.2**	EcoRI/XhoI	GAATTCATGGCTTCTTCTTCAACCAAGAAAGC	CTCGAGCTACTCCACCTTGCTAATCCCAATC

**Y2H-AD-FaMYB44.1**	EcoRI/BamHI	GAATTCATGGCTATGAATCGGAAGGA	GGATCCTCACTCAATTCTGCTGATC
**Y2H-AD-FaMYB44.2**	NdeI/EcoRI	CATATGATGGCTTCTTCTTCAACCAG	GAATTCCTACTCCACCTTGCTAATCC
**Y2H-BD-FaMYB44.2**	NdeI/EcoRI	CATATGATGGCTTCTTCTTCAACCAG	GAATTCCTACTCCACCTTGCTAATCC
**Y2H-AD-FaMYB44.3**	NdeI/EcoRI	CATATGATGGATTGCGAATCGGTTCAGCAGA	GAATTCTTACGAGAGCTTGGGTATACCAAGG
**Y2H-AD-FabHLH3**	EcoRI/BamHI	GAATTCATGGGTGAGAAGCTTTGGGTGAAT	GGATCCTTACACGGATGATTTTGAAAGAGC
**Y2H-BD-FabHLH3**	EcoRI/BamHI	GAATTCATGGGTGAGAAGCTTTGGGTGAAT	GGATCCTTACACGGATGATTTTGAAAGAGC
**Y2H-AD-FabHLH33**	NdeI/EcoRI	CATATGATGCGGATAATTGAAAACCCATGCT	GAATTCTTAACACTTACCAGCAATTTTCCAA
**Y2H-BD-FabHLH33**	NdeI/EcoRI	CATATGATGCGGATAATTGAAAACCCATGCT	GAATTCTTAACACTTACCAGCAATTTTCCAA
**Y2H-AD-FaTTG1**	NdeI/XmaI	CATATGATGGAGAATTCGACCCTCGAATCCCACC	CCCGGGTCAAACCTTCAAGAGCTGCATCTTGTT
**Y2H-BD-FaTTG1**	NdeI/XmaI	CATATGATGGAGAATTCGACCCTCGAATCCCACC	CCCGGGTCAAACCTTCAAGAGCTGCATCTTGTT
**Y2H-AD-FaMYB1**	NdeI/XmaI	CATATGATGAGGAAGCCCTGCTGCGAGA	CCCGGGTTAAGCAACTTGAGGATCAGCCA
**Y2H-AD-FaMYB10**	EcoRI/BamHI	GAATTCATGGAGGGTTATTTCGGTGTG	GGATCCTCATACGTAGGAGATGTTGACTAGAT
**PMDC83-FaMYB44.2**	SpeI/AscI	ACTAGTATGGCTTCTTCTTCAACCAAGAAAGC	GGCGCGCCTCTCCACCTTGCTAATCCCAATC
**SPYCE- FaMYB44.1**	SpeI/XhoI	ACTAGTATGGCTATGAATCGGAAGGAAAT	CTCGAGCTCAATTCTGCTGATCCCAATTC
**SPYNE- FaMYB44.2**	SpeI/SalI	ACTAGTATGGCTTCTTCTTCAACCAAGAA	GTCGACCTCCACCTTGCTAATCCCAATCC
**SPYCE- FaMYB44.2**	SpeI/SalI	ACTAGTATGGCTTCTTCTTCAACCAAGAA	GTCGACCTCCACCTTGCTAATCCCAATCC
**SPYCE- FaMYB44.3**	SpeI/XhoI	ACTAGTATGGATTGCGAATCGGTTCAGCAGA	CTCGAGCGAGAGCTTGGGTATACCAAGGTGC
**SPYNE-FaMYB10**	BamHI/SmaI	GGATCCATGGAGGGTTATTTCGGTGTGAGA	CCCGGGGGAGATGTTGACTAGATC
**SPYCE-FaMYB10**	BamHI/SmaI	GGATCCATGGAGGGTTATTTCGGTGTGAGA	CCCGGGGGAGATGTTGACTAGATC
**SPYCE- FaMYB1**	BamHI/SmaI	GGATCCATGAGGAAGCCCTGCTGCGAGA	CCCGGGAGCAACTTGAGGATCAGCCATTC
**SPYCE-FabHLH3**	SpeI/XhoI	ACTAGTATGGGTGAGAAGCTTTGGGTGAAT	CTCGAGCACGGATGATTTTGAAAGAGCAGC
**SPYCE-FabHLH33**	BamHI/SmaI	GGATCCATGCGGATAATTGAAAACCCATGCT	CCCGGGACACTTACCAGCAATTTTCCAAAGC

**SPYCE-FaTTG1**	BamHI/SmaI	GGATCCATGGAGAATTCGACCCTCGAATCC	CCCGGGAACCTTCAAGAGCTGCATCTTGTT
**SPYCE-FaMADS9**	BamHI/SmaI	GGATCCATGGGGAGGGGAAGAGTGGAGC	CCCGGGGAGCATCCAACCAGGAATGAAA
**nLUC-FaMYB44.2**	SacI/SalI	GAGCTCATGGCTTCTTCTTCAACCAAGA	GTCGACCTCCACCTTGCTAATCCCAATCC
**cLUC-FaMYB44.2**	SalI/PstI	GTCGACATGGCTTCTTCTTCAACCAAGAAAG	CTGCAGCTACTCCACCTTGCTAATCCCAATC
**cLUC-FabHLH3**	KpnI/BamHI	GGTACCATGGGTGAGAAGCTTTGGGTGAAT	GGATCCTTACACGGATGATTTTGAAAGAGC
**cLUC-FaTTG1**	KpnI/BamHI	GGTACCATGGAGAATTCGACCCTCGAATC	GGATCCTCAAACCTTCAAGAGCTGCATC

Table S3 Primers used for gene expression analysis by qRT-PCR.

Gene	Full Name	Gene ID	Forward primer (5'-3')	Reverse primer (5'-3')	Note (related ripening parameter)
FaMYB44.1	MYB domain protein 44.1	gene24027	ATCCTCCAACTTCGCTGTCC	GCCTGGTTCTGAGTCGACTC	——
FaMYB44.2	MYB domain protein 44.2	gene00185	CTCATTCGCAAGCAGCCTTC	GCTGAGTGATGTCGGTGGAT	Sugar and acid accumulation
FaMYB44.3	MYB domain protein 44.3	gene26289	GCGCATTCGAAATACGGCAA	CATGGACGAGTACTTCCGCT	——
FaSUS1	Sucrose synthase 1	gene12940	CCCTGATTCTGACCTTTACTGG	GATGATGAAGTCGGTGTGGTT	Sugar metabolism
FaSUS2	Sucrose synthase 2	gene11077	TTGCTGAGGATGTTGCAGGT	AACAACGAGGCGACGAGATT	Sugar metabolism
FaSPS1	Sucrose-phosphate synthase 1	gene11606	GAGCCTTGAATGTCCCAATGT	ATCTCCTGTCTGGTGCTGGTT	Sugar metabolism
FaSPS2	Sucrose-phosphate synthase 2	gene22863	ACTGTGCAAAGTTGGCGTCTT	GATCATCGCAATCCACGGCTA	Sugar metabolism
FaSPS3	Sucrose-phosphate synthase 3	gene31122	CGTAGATTGGAGTTATGGAGAGC	CGAATGATGTAAGAACCACTGC	Sugar metabolism
FaSUT1	Sucrose transport protein 1	gene26850	GAGTGTTTGTGTTTGGGTTTTG	CGATGGTCCTTTCCAGTGA	Sucrose transporter
FaNI	Neutral invertase	gene05019	CGACCTCGGTGGATAGTCAG	CCACATCACCATTCCCCGTC	Sugar metabolism
FaHXK1	Hexokinase 1	gene11313	AGCATTGGATGGTGGCTTGT	TATGTGCTCGGCGACATCAT	Sugar metabolism and signaling

FaHXK2	Hexokinase 2	gene08515	GCTGTCACCGAGCTTCTAGG	CAGCCCCTATACCAGACCCA	Sugar metabolism and signaling
FaTPS7	Trehalose phosphate synthase 7	gene29717	CTGCTGTGAGGGATGGGATG	CTTGGGCCCACTGGATTCTG	Sugar metabolism and signaling
FaFRK4	Fructokinase 4	gene21673	GCCTTGCTCTCCTATGACCC	TGACCTTGATCACGTCTGCC	Sugar metabolism and signaling
FaCS1	Citrate synthase 1	gene00583	ATCCTGATGCCAATCCTGCC	TGGTGCCTTCCCAAGAATCC	Citric acid accumulation
FaCS2	Citrate synthase 2	gene05118	CATCCCACTGCCGATGTCTT	GACTCTGATGGTTGGCTGCT	Citric acid accumulation
FaVHA-A	V-type proton ATPase catalytic subunit A	gene05361	AGAGCGAGTTTGGCTACGTC	CATTTCCGACACGAACCAGC	Acid metabolism
FaMDH1	Malate dehydrogenase 1	gene05605	GGTGTTCAAGAAAGCCGGGA	CCCAGAACCTCTGCCACAAA	Malic acid metabolism
FaMDH2	Malate dehydrogenase 2	gene11464	TGTGACCACATCCGGGATTG	AGCTGGTACACCGTATGAGC	Malic acid metabolism
FaGAD	Glutamate decarboxylase	gene09305	GCCTACACTATGCCAGCCAA	GAGTCTCTCTGCCAAGCTCC	Acid metabolism
FaNADP-ME	NADP-dependent malic enzyme	gene11286	TGGTGACCTTGGTTGTCAGG	GGCAAACATGTTGAGGGACG	Malic acid metabolism
FaAco	Aconitate hydratase	gene23775	CCTTACACGCTTCGCTCTCA	ATGACTTTCCGGAGCCATGG	Acid metabolism
FaVHP	Vacuolar membrane proton pump	gene30771	GTGGGAAGTGCTGCTCTGAA	AGCATAGTCAGGCTTGGCAG	Acid metabolism
FaAMY3	Alpha-amylase 3	gene04016	TAGCTGCGACGTTTGAGTCC	CCCGTGATCCAACCTCACAT	Starch metabolism
FaBAM1	Beta-amylase 1	gene28316	GGGACATGGTCATTCCCTGG	GTGCTACCCCATTCTGGCTT	Starch metabolism
FaFUM1	Fumarate hydratase 1	gene11092	GGACGTACTCACACGCAAGA	TGCACAAGACTCGCTCGATT	Starch metabolism
FaADSS	Adenylosuccinate synthase	gene03239	GGTGTTGTGGTGCATCTTCC	GGTGAGCACGATCAGAGACC	Starch metabolism
FaQR	Quinone oxidoreductase	gene28406	CACTGACTCTCCCCTACCTACA	ATACACTTCATCCCCCACCTTA	Aroma production
FaEXP1	Expansin 1	gene18424	AGGACGGAGTTGGATTGC	TGAGCGTGAGCGTGAAG	Softness

FaEXP3	Expansin 3	gene04435	TCACTGCCACTA ACTTCTGC	TTATGCCTCCTGC TCTCCT	Softness
FaXYL1	Xylosidase 1	gene05164	ATGGAAAGCCT ACTTGTGCTG	CTGGTGTAATGTT GTTGGTCGT	Softness
FaCHS	Chalcone synthase	gene26825	GCCTTTGTTTGG CTGGCT	CCCAGGAACATCT TTGAGGA	Anthocyanin accumulation
FaCHI	Chalcone--flavonone isomerase	gene23367	AGCGAAAGCCA TTGAAAAGT	CATTTGGTGATTG TGTGAAGAG	Anthocyanin accumulation
FaPAL	Phenylalanine ammonia-lyase	gene23261	CTTCTGTGGTGC TGTTTGATG	AGGGTGGTGCTTC AGTTTATGT	Flavonoid metabolism
FaMADS9	MADS-box transcription factor 9	gene04229	ACGCTGAGGTT GCTCTCATC	AGGACTCTAGCTG ACGCTCA	Ripening-related transcription factor
FaSHP	SHATTERPR OOF-like	gene24494	TGATCCTGAGA GCTCTTCCCA	GCGCTTGCAGAAT GTGACTT	Ripening-related transcription factor
	MADS-box transcription factor	gene04563	GAGAAGGAACG GGTTGCTGA	ACTCATAGAGCTT GCCACGG	
	MADS-box transcription factor	gene26118	GTGCTCCACTTC ATCTGGCA	TGCTGAAAGAAA CCTTGCGAC	
FaMYB1	MYB domain protein 1	gene09407	TAGCTGGAAGA CTGCCTGGA	GGACGAAGAGTA GTGCCTGT	Anthocyanin accumulation
FaMYB10	MYB domain protein 10	gene31413	CAACAGCACCA CCACAGACT	GCTTGCCGATTGT ACCGTAT	Anthocyanin accumulation
FaEOBII	Emission of benzenoid II	gene28435	GCAGATCAGGC AAGCACAAG	GAGGCTGAACCA GAGTTGCT	Aroma production
FaGAMYB	Transcription factor GAMYB	gene00431	ATCCAATGTGAA ACCTGAACCA	CCAGCCAATCTGA ACCAAGTAA	Anthocyanin accumulation
FabHLH3	Basic helix-loop-helix 3	gene08231	CAGGAGTTGTG GAGCTTGGT	GCCTGGACAGAG CTAGGAAC	Ripening-related transcription factor
FabHLH33	Basic helix-loop-helix 33	gene19321	TGACAAGGCAT CGATCCTCG	GTGCTTCCACAGT GTCCATG	Ripening-related transcription factor
FaTTG1	Transparent testa glabra 1	gene12450	GGACTTGAGGT ACATGGCGA	ATTCACACTCCCT CTGTGCC	Ripening-related transcription factor
FaCOI1	Coronatine-insensitive protein 1	gene20913	GAGGAGATTGC GGAGTCGTT	CGGAGCACTTGTC CAGCTTA	JA receptor
FaJAZ1	Jasmonate-zim-domain protein 1	gene12541	AACCTCGTCGA CATGCAGTT	ATTTGAAGCCTCT GGGAGCC	JA signaling

FaJAZ2	Jasmonate-zim-domain protein 2	gene00583	AGACATGCAACCTCCTCAGC	GATCTCCTTGGCCTTCTCCG	JA signaling
FaJAZ8	Jasmonate-zim-domain protein 8	gene30624	ATTTGGAGCTCCGCCTTAAT	GAAGATCGTCAATGGCTGCT	JA signaling
FaMYC2	Transcription factor MYC2	gene10501	CTCTCCGAGCTGTTGTTCCA	TCTGACTCCGTCGTTTGCAG	JA signaling
FaOPR3	Oxophytodienoate reductase 3	gene12480	GGAGCCGTTATTTTCTGTCAGC	TCAAAACCTGCTCGAATGGC	JA biosynthesis
FaARF6A	Auxin response factor 6A	gene22728	TGAAGTGCAACAAATGGGAA	CGGCTTACGTAGTCGTCACA	Auxin signaling
FaARF6B	Auxin response factor 6B	gene30394	TGGCTTGCAGACAGACATGT	GCAGAAGGGACGTAGGTAGC	Auxin signaling
FaPIN8	Auxin efflux carrier component 8	gene16792	ACCCTCCCACTCTTCACCTT	GCACAACCACGATGATGAGC	Auxin transporter
FaMRLK47	FERONIA/FER-like receptor kinase 47	gene13568	GCTTAGGTCTTGGTATGATGA	TAGTTGAGGTTGATGTTGTTATTC	Ripening-related protein kinase
FaPYL1	Pyrabactin resistance like 1	gene10508	GCTTCGTAATGAGCGTGGGA	CTCTCCTCGTCCAGCAAGTC	ABA receptor
FaPYL8	Pyrabactin resistance like 8	gene13100	GGAGGAGGTGCGAGTGAGTA	TGAACAGGCGCTTTGATGTG	ABA receptor
FaPYL9	Pyrabactin resistance like 9	gene19210	ACTGGAGCTTCTGGATGACG	ACCTCAGGATGGACGGTGAT	ABA receptor
FaSnRK2.6	SNF1-related protein kinase2.6	gene31902	GCTACACTCGCAACCAAAATC	ACCCCACAAGACCAGACATC	Ripening-related protein kinase
FaNCED1	9-cis-epoxycarotenoid dioxygenase 1	gene31335	ACTGCTTCTGCTTCCATCTCT	AGACACTCGTCGCATTCATT	ABA biosynthesis
FaNCED2	9-cis-epoxycarotenoid dioxygenase 2	gene30616	GACACCTTTTGCTTCCACTTG	GGATTTCGGACAACACACTCT	ABA biosynthesis

FaNCED3	9-cis-epoxycarotenoid dioxygenase 3	gene24386	CAAACCCTATCACCCTTCTCAC	TGGGCAACTTCTGCTTCTTT	ABA biosynthesis
FaACTIN	Actin protein	gene22626	GCCAACCGTGAGAAGATG	TCCAGAGTCAAGAACAATACCAG	——

Table S4. Primers used to construct the vectors for the GUS activity assay. The enzyme sites in the primer sequences are underlined.

**Primer name**	**Enzyme sites**	**Forward primer (5'-3')**	**Reverse primer (5'-3')**
**PBI121-FaMYB10**	XbaI/SacI	TCTAGAATGGAGGGTTATTTCGGTGTG	GAGCTCGGATCCTCATACGTAGGAGATGTTGACTAGAT
**1304- P$_{FaSPS3}$**	NcoI/BglII	CCATGGTTAGCGTCTACTCTCCACAGTTTCCTGCC	AGATCTCGTCGGAGCTCGAATTCAGACCAAATC
**1304-FaMYB44.2**	EcoRI/XhoI	GAATTCATGGCTTCTTCTTCAACCAAGAAAGC	CTCGAGCTACTCCACCTTGCTAATCCCAATCC
**1304-FabHLH3-S**	PstI/BamHI	CTGCAGATGGGTGAGAAGCTTTGGGTGAATGA	GGATCCTTACACGGATGATTTTGAAAGAGCAG
**1304-FabHLH3-D**	PstI/SalI	CTGCAGATGGGTGAGAAGCTTTGGGTGAATGA	GTCGACTTACACGGATGATTTTGAAAGAGCAG
**1304-FaTTG1-S**	HindIII/XbaI	AAGCTTATGGAGAATTCGACCCTCGAATCC	TCTAGATCAAACCTTCAAGAGCTGCATC
**1304-FaTTG1-D**	BamHI/KpnI	GGATCCATGGAGAATTCGACCCTCGAATCC	GGTACCTCAAACCTTCAAGAGCTGCATC

Table S5. Probes used for the EMSA. The sequences of cis-elements are underlined.

**Primer name**	**Forward primer (5'-3')**	**Reverse primer (5'-3')**
**P$_{FaSPS3}$-MBS**	ATTGCATTTTAGTTTTCAACTGTTTTACGTGTTTAGTCAG	CTGACTAAACACGTAAAACAGTTGAAAACTAAAATGCAAT
**P$_{FaSPS3}$-CGTCA-motif**	ATCAATTTTCTTTGGAATGACGAGTGTATGGTAAACTAAA	TTTAGTTTACCATACACTCGTCATTCCAAAGAAAATTGAT
**P$_{FaSPS3}$-TGA-element**	TTAAAACTAGGGTGGAAACGACAGAGATAAGGCGTTTACG	CGTAAACGCCTTATCTCTGTCGTTTCCACCCTAGTTTTAA
**P$_{FaCS}$-MBS**	TGTGGTGAACATAACTGCTTTCTTAATTGATTACTTTAAA	TTTAAAGTAATCAATTAAGAAAGCAGTTATGTTCACCACA
**P$_{FaSUS1}$-HSE+TC-rich**	GTGGGTAAGAGAAAATTCATAGTCTTCTGGGAAGAAAATATAGTATATAT	ATATATACTATATTTTCTTCCCAGAAGACTATGAATTTTCTCTTACCCAC
**P$_{FaSUS1}$-TC-rich**	CCAAGTCTTTGCATTTACTCCATTTCTGTTTCGCGCTTCT	AGAAGCGCGAAACAGAAATGGAGTAAATGCAAAGACTTGG

**P*FaSUS1*-TATCCAT**	TATACAGAAAAGGAGAGTATCCATGGATGGACATGCAAGA	TCTTGCATGTCCATCCATGGATACTCTCCTTTTCTGTATA
**P*FaHXK2*-G-box**	AATAAAAACCAACGTGTTGATCTCATACTTCATTTTCGTAGATCTCCACGTAATAACTG	CAGTTATTACGTGGAGATCTACGAAAATGAAGTATGAGATCAACACGTTGGTTTTTATT
**P*FaHXK2*-ABRE**	TTCGTAGATCTCCACGTAATAACTGTAACC	GGTTACAGTTATTACGTGGAGATCTACGAA
**P*FaHXK2*-MBS**	TCCACGTAATAACTGTAACCGTACGTCAACCAACTCAGCA	TGCTGAGTTGGTTGACGTACGGTTACAGTTATTACGTGGA
**P*FaHXK2*-CGTCA-motif**	TAGTAGCTGCGCTTCCGTCATGCTCCATGGACCAATGACGATCGTAAACT	AGTTTACGATCGTCATTGGTCCATGGAGCATGACGGAAGCGCAGCTACTA
**P*FaHXK2*-TATCCAT**	TCACTATGTCAAATGAAACTATCCATGGGCGGCAACTCGA	TCGAGTTGCCGCCCATGGATAGTTTCATTTGACATAGTGA
**P*FaMDH1*-MBS**	TTCTTGCTTTCTGAAACTGATAACTGATTCAAATTGTGAT	ATCACAATTTGAATCAGTTATCAGTTTCAGAAAGCAAGAA
**P*FaMDH1*-TATCCAT**	TTTATGTCTGATAACGTGGTTTATCCATGACATTGTATTT	AAATACAATGTCATGGATAAACCACGTTATCAGACATAAA

Table S6. Sequences and primers used for microRNA expression analysis by qRT-PCR.

miRNA family	miRNA name	Forward primer (5'-3')	Sequence
**miR159**	**fve-miR159a**	ATTGGATTGAAGGGAGCTCTC	AUUGGAUUGAAGGGAGCUCUC
**miR159b**	**fve-miR159b**	CGTTGGATTGAAGGGAGCTCTA	UUUGGAUUGAAGGGAGCUCUA
**miR828**	**fve-miR828**	GCCTCTTGCTTAAATGAGTATTCCA	UCUUGCUUAAAUGAGUAUUCCA
**miR156**	**fve-miR156a**	GCTTGACAGAAGAGAGTGAGCAC	UUGACAGAAGAGAGUGAGCAC
**miR156**	**fve-miR156h**	GCCTGACAGAAGAGAGTGAGCTC	UGACAGAAGAGAGUGAGCUC
**miR399**	**fve-miR399a**	TGCCAAAGGAGAGTTGCCCT	UGCCAAAGGAGAGUUGCCCUG
**miR319**	**fve-miR319a**	CTTGGACTGAAGGGTGCTCCTT	CUUGGACUGAAGGGUGCUCCUU
**U6**		GGGGACATCCGATAAAATT	

Table S7. Primers used for gene expression analysis by RT-PCR.

Gene	Full Name	Gene ID	Forward primer (5'-3')	Reverse primer (5'-3')
FaSUS1	Sucrose synthase 1	gene12940	TCCGATATTCTTCGTGTTCCC	TGATGTTCTTTACACGGTCCA
FaSUS2	Sucrose synthase 2	gene11077	GAATGATCCATCAAAGCCC	AGACATACTTCCAGAAACCGTA
FaSPS3	Sucrose-phosphate synthase 3	gene31122	CCGCATGGTGGTAATTCCTC	CCCTTTGTCTTTGCTGCGAGA
FaMYB44.2	MYB domain protein 44.2	gene00185	TCAAGCGCAAATTCTCGTCCATGTCC	GAACTGCTTATCAATCCCCACCTCACT

FaMADS9	MADS-box transcription factor 9	gene04229	ATGGGGAGGGGAAGAGTGG AGC	GAGCATCCAACCAGGAATGAAA
FaMYB10	MYB domain protein 10	gene31413	ATGGAGGGTTATTTCGGTGT G	TCATACGTAGGAGATGTTGACTA GAT
FaACTIN	Actin protein	gene22626	TGGGTTTGCTGGAGATGAT	CAGTTAGGAGAACTGGGTGC

Table S8. *FaSUS, FaSPS and FabHLH33* expression at different developmental and ripening stages according to RNA-sequence data. The RNA-seq was performed with diploid strawberry (*Fragaria* × *ananassa* 'Benihoppe'). RPKM (Reads Per Kilobase Million) value was used to present the gene expression levels, while the changed fold of genes was calculated using the log2 ratio of RPKM value between two different samples. Gene ID of FaSUSs and FaSPSs were obtained using the protein sequences of AtSUS and AtSPS as queries BLAST against the strawberry genome database.

**Gene Description**		**Expression Level (RPKM)**			**Fold Change (Log_2*)**	
**Gene Name**	**Gene ID**	**Green fruit**	**White fruit**	**Red fruit**	**White/Green***	**Red/White***
**Sucrose synthase 1**	gene12940	213.37	146.73	48.83	-0.54	-1.58
**Sucrose synthase 2**	gene11077	37.68	44.91	74.31	0.25	0.72
**Sucrose synthase 3**	gene07050	1.75	0.76	0.05	-1.19	-3.89
**Sucrose synthase 4**	gene11429	5.89	6.14	3.73	0.06	-0.71
**Sucrose synthase 5**	gene31666	6.64	5.36	0.87	-0.30	-2.62
**Sucrose-phosphate synthase 1**	gene11606	0.65	0.56	4.68	-0.22	3.06
**Sucrose-phosphate synthase 2**	gene22863	7.89	14.38	15.23	0.86	0.08
**Sucrose-phosphate synthase 3**	gene31122	52.34	91.67	156.54	0.81	0.77
**Sucrose-phosphate synthase 4**	gene06523	1.62	1.43	0.40	-0.17	-1.83
**Sucrose-phosphate synthase 5**	gene31164	19.03	11.61	9.41	-0.71	-0.30
**Sucrose-phosphate synthase 6**	gene04429	11.54	12.67	7.65	0.13	-0.72
**Sucrose-phosphate synthase 7**	gene03841	0.13	0.04	0.17	-1.69	2.04
**Sucrose-phosphate synthase 8**	gene04408	9.34	10.96	14.37	0.23	0.39

**FabHLH33**	gene19321	0.10	0.18	0.88	0.75	0.07

Table S9. Quantitative analysis of *FaMYB10* and *FaMYB44.2* expression levels by qRT-PCR. The $2^{-\Delta\Delta CT}$ method was used to calculate transcript levels (Schmittgen and Livak, 2008). Transcript values were normalized using *FaACTIN* as an internal control. The ΔCT of small green fruit was chosen as the calibrator (Livak & Schmittgen, 2001). The ratio of gene expression levels in different fruit stages between FaMYB10 and FaMYB44.2 was calculated by transcript values. Three biological replicates were repeated.

Fruit Stage	Ratio of gene expression levels
Green fruit	*FaMYB10*: *FaMYB44.2* = 0.16
White fruit	*FaMYB10*: *FaMYB44.2* =4.67
Turning fruit	*FaMYB10*: *FaMYB44.2* = 6.28
Red fruit	*FaMYB10*: *FaMYB44.2* = 13.22
